# Overexpression of Human Syndecan-1 Protects against the Diethylnitrosamine-Induced Hepatocarcinogenesis in Mice

**DOI:** 10.3390/cancers13071548

**Published:** 2021-03-27

**Authors:** Andrea Reszegi, Katalin Karászi, Gábor Tóth, Kristóf Rada, Lóránd Váncza, Lilla Turiák, Zsuzsa Schaff, András Kiss, László Szilák, Gábor Szabó, Gábor Petővári, Anna Sebestyén, Katalin Dezső, Eszter Regős, Péter Tátrai, Kornélia Baghy, Ilona Kovalszky

**Affiliations:** 1 1st Department of Pathology and Experimental Cancer Research, Semmelweis University, Üllői út 26, H-1085 Budapest, Hungary; reszegi.andrea@med.semmelweis-univ.hu (A.R.); karaszi.katalin@med.semmelweis-univ.hu (K.K.); rada.kristof@koki.hu (K.R.); vancza.lorand@med.semmelweis-univ.hu (L.V.); petovari.gabor@med.semmelweis-univ.hu (G.P.); sebestyen.anna@med.semmelweis-univ.hu (A.S.); dezso.katalin@med.semmelweis-univ.hu (K.D.); regos.eszter@med.semmelweis-univ.hu (E.R.); baghy.kornelia@med.semmelweis-univ.hu (K.B.); 2 MS Proteomics Research Group, Research Centre for Natural Sciences, Eötvös Loránd Research Network, Magyar Tudósok Körútja 2, H-1117 Budapest, Hungary; toth.gabor@ttk.hu (G.T.); turiak.lilla@ttk.hu (L.T.); 3 Department of Endocrine Neurobiology, Institute of Experimental Medicine, Hungarian Academy of Sciences, Szigony utca 43, H-1083 Budapest, Hungary; 4 2nd Department of Pathology, Semmelweis University, Üllői út 93, H-1091 Budapest, Hungary; schaff.zsuzsa@med.semmelweis-univ.hu (Z.S.); kiss.andras@med.semmelweis-univ.hu (A.K.); 5 Szilak Laboratories Bioinformatics and Molecule-Design Ltd., Gem utca 14, H-6723 Szeged, Hungary; ughy@brc.hu; 6 Medical Gene Technology Unit, Institute of Experimental Medicine, Hungarian Academy of Sciences, Szigony utca 43, H-1083 Budapest, Hungary; szabog@koki.hu; 7 Solvo Biotechnology, Irinyi József utca 4-20, H-1117 Budapest, Hungary; peter.tatrai@crl.com

**Keywords:** syndecan-1, liver carcinogenesis, mouse, lipid metabolism

## Abstract

**Simple Summary:**

Syndecan-1 is a Janus-faced proteoglycan: depending on the type of cancer, it can promote or inhibit the development of tumors. Our previous in vitro experiments revealed that transfection of human syndecan-1 (hSDC1) into hepatoma cells, initiating hepatocyte-like differentiation. To further confirm the antitumor action of hSDC1 in the context of liver carcinogenesis, mice transgenic for albumin promoter-driven hSDC1 were created with exclusive expression of hSDC1 in the liver. Indeed, hSDC1 interfered with the development of liver cancer in diethylnitrosamine (DEN)-induced hepatocarcinogenesis experiments. The mechanism was found to be related to lipid metabolism that plays an important role in the induction of nonalcoholic liver cirrhosis. Nonalcoholic fatty liver disease is known to promote the development of cancer; therefore, the oncoprotective effect of hSDC1 may be mediated by a beneficial modulation of lipid metabolism.

**Abstract:**

Although syndecan-1 (SDC1) is known to be dysregulated in various cancer types, its implication in tumorigenesis is poorly understood. Its effect may be detrimental or protective depending on the type of cancer. Our previous data suggest that SDC1 is protective against hepatocarcinogenesis. To further verify this notion, human SDC1 transgenic (hSDC1^+/+^) mice were generated that expressed hSDC1 specifically in the liver under the control of the albumin promoter. Hepatocarcinogenesis was induced by a single dose of diethylnitrosamine (DEN) at an age of 15 days after birth, which resulted in tumors without cirrhosis in wild-type and hSDC1^+/+^ mice. At the experimental endpoint, livers were examined macroscopically and histologically, as well as by immunohistochemistry, Western blot, receptor tyrosine kinase array, phosphoprotein array, and proteomic analysis. Liver-specific overexpression of hSDC1 resulted in an approximately six month delay in tumor formation via the promotion of SDC1 shedding, downregulation of lipid metabolism, inhibition of the mTOR and the β-catenin pathways, and activation of the Foxo1 and p53 transcription factors that lead to the upregulation of the cell cycle inhibitors p21 and p27. Furthermore, both of them are implicated in the regulation of intermediary metabolism. Proteomic analysis showed enhanced lipid metabolism, activation of motor proteins, and loss of mitochondrial electron transport proteins as promoters of cancer in wild-type tumors, inhibited in the hSDC1^+/+^ livers. These complex mechanisms mimic the characteristics of nonalcoholic steatohepatitis (NASH) induced human liver cancer successfully delayed by syndecan-1.

## 1. Introduction

Syndecan-1 (SDC1), a transmembrane proteoglycan acting as an auxiliary cell surface receptor, is critical in establishing connections between the extracellular matrix and intracellular compartments. Its heparan sulfate (HS) and chondroitin sulfate glycosaminoglycan chains associate with a plethora of extracellular ligands and promote binding to their high-affinity receptors. The interactions of SDC1 with tyrosine kinase (TK) receptors or their ligands initiate or modulate inward-directed signals. SDC1 can establish connections with various integrins, thereby creating ternary signaling complexes with TK receptors and ligands. The cytoplasmic domain of SDC1 cooperates with several intracellular proteins including cortactin, PKC, paxillin, alpha-actinin, and FAK, as well as proteins bearing PDZ domains, among others [1,2,3,4]. SDC1 also actively participates in calcium signaling [5].

Although SDC1 is still primarily thought of as a cell surface protein, evidence has been mounting in the past two decades showing that full-length or truncated forms of SDC1 may reside in the cytoplasm, or even in the nucleus, where they may exert additional and previously unexpected functions [6,7,8,9].

In addition to an already broad range of physiological roles, SDC1 is also implicated in a multitude of pathological processes, including inflammation, wound healing, and cancer. Notably, reports on its role in cancer are conflicting, with both pro- and anti-tumorigenic potential proposed in different tumor types [10].

Although only a relatively low amount of SDC1 is found on the basolateral surface of hepatocytes, SDC1 is still the major proteoglycan (PG) in the healthy liver. It contributes to the hepatic clearance of low-density lipoprotein [11] and is also known as a receptor of hepatitis virus C [12]. These receptor functions of SDC1 are mostly attributed to its HS chains that associate with growth factors and cytokines, bind remnant lipoprotein, and sequester hepatitis viruses. SDC1–ligand interactions can promote signaling inside hepatocytes but may also remove molecules from the cell surface via shedding of SDC1. Shedding is a specific function of SDC1 that modulates the availability of critical factors bound to the proteoglycan [13,14].

We reported that SDC1 expression is upregulated in hepatic inflammation and consecutive fibrosis, resulting in a circumferential pattern of SDC1 immunostaining on the surface of hepatocytes, and the deterioration of liver function is accompanied by enhanced SDC1 shedding [15]. Intriguingly, while the expression of SDC1 increases in human hepatocellular cancer (HCC) that develops in the cirrhotic liver, SDC1 becomes suppressed in HCC formed in a non-cirrhotic background. The processes of fibrogenesis and hepatocarcinogenesis run concomitantly in cirrhosis-associated HCC; therefore, the overexpression of SDC1 may be more related to cirrhosis than to carcinogenesis, and high expression of SDC1 may ultimately counter rather than support tumor growth. Consistent with this notion, overexpression of full-length or extracellular domain-truncated syndecan-1 in human hepatoma cell lines resulted in cell differentiation via downregulation of the transcription factors Ets-1 and AP-1 [16].

To explore the potentially beneficial role of SDC1 in liver carcinogenesis, we established a human syndecan-1 transgenic (hSDC1^+/+^) mouse model that expresses hSDC1 specifically in hepatocytes under the control of the albumin promoter. Wild-type (WT) and hSDC1^+/+^ mice were exposed to diethylnitrosamine (DEN)-induced hepatocarcinogenesis to investigate whether targeted overexpression of syndecan-1 in the liver is capable of delaying the development of hepatocellular cancer.

## 2. Materials and Methods

### 2.1. Generation of Human Syndecan-1-Transgenic (hSDC^+/+^) Mice

All animal studies were performed according to the ethical standards of the Animal Health Care and Control Institute, Csongrád County, Hungary (ethical license: XVI/03047-2/2008).

To generate a mouse strain that overexpresses human syndecan-1 (hSDC1) in the liver, we designed a vector containing human syndecan-1 cDNA driven by the mouse albumin promoter (mAlb). A 3.6 kb DNA fragment was isolated from the mAlb/hSDC1 plasmid with SalI-EciI digestion and subsequent electroelution. Fragments were purified and microinjected into inseminated FVB/N mouse oocytes, and the oocytes were transferred into CD1 host females. Offspring were tested for the presence of the targeted gene. DNA isolated from progeny was digested with EcoRI enzyme, which split an approximately 2.2 kb fragment with the HindIII sequence used as a probe. The final product was detected on a 1% *w*/*v* Tris–Borate–EDTA agarose gel. The forward (F) and reverse (R) primers for genotyping were as follows (F: 5′–GGC TGT AGT CCT GCC AGA AG–3′) and (R: 5′–GTA TTC TCC CCC GAG GTT TC–3′). After genotyping, the transgene was found in one male and two females. Transgenic animals were backcrossed into the FVB/N background for nine generations until homozygosity. Animals were produced in the Institute of Experimental Medicine of the Hungarian Academy of Sciences. The expression of hSDC1 was confirmed by fluorescence immunohistochemistry, as described before [17].

**Figure 1:**
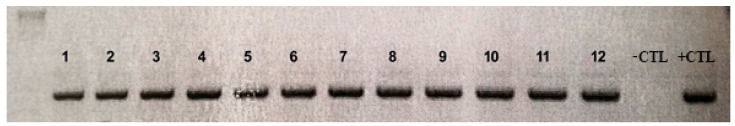
The homozygous presence of hSDC1 DNA by PCR from the tails of 12 C57 Black offspring. -CTL: non-template control; +CTL: parental transgenic animal.

Livers of the FVB/N mouse strain proved to be resistant to DEN hepatocarcinogenesis; therefore, we generated C57 Black transgenic animals by repeated backcrossing through 9 generations again until no hSDC1-negative descendant was born. The presence of human syndecan-1 was followed by PCR using DNA isolated from the tail of the mice (Table 1) (Figure 1).

### 2.2. Hepatocarcinogenesis

Hepatocarcinogenesis was induced by a single high-dose (15 μg/g body weight) intraperitoneal injection of DEN at the age of 15 days. Forty DEN-exposed hSDC1^+/+^ mice (hSDC1^+/+^ DEN) and 43 untreated hSDC1^+/+^ controls (hSDC1^+/+^ CTL), as well as 26 DEN-exposed wild-type C57/Black (WT DEN) and 16 untreated C57/Black (WT CTL) mice, were compared (Table 2). DEN-induced hepatocarcinogenesis is one of the most frequently applied models to study the development of liver cancer. DEN, metabolized and activated by cytochrome p450 enzymes, forms adducts with DNA and induces random mutations [18]. Young mice exposed to a single dose of the mutagen at the age of 15 days rapidly and reproducibly develop hepatocellular cancer. DEN exposure does not induce liver cirrhosis; thus, cancer develops in non-cirrhotic liver. Development of liver tumors was followed up at 3, 6, and 9 months after DEN exposure. Observation was extended to month 11 for hSDC1^+/+^ DEN mice where tumor formation was substantially delayed. At termination, body and liver weights of the animals were measured and macroscopically detectable tumors were counted. Half of the liver samples was fixed in 10% formaldehyde and embedded in paraffin for histological analysis; the other half was frozen for further analyses. Formalin-fixed paraffin-embedded sections (FFPE) were stained with hematoxylin and eosin (HE) or processed for immunohistochemistry. Stained sections were used for histological diagnosis. HE-stained sections were scanned by Pannoramic Scan (3DHistech Ltd., Budapest, Hungary); the length and width of tumors in each section were determined using Pannoramic Viewer (3DHistech Ltd.); and tumor volume was calculated as V = width (mm)^2^ × length (mm) × π/6.

### 2.3. ELISA

To determine TGF-β1 levels, frozen liver samples were extracted in lysis buffer (20 mM Tris pH 7.5, 2 mM EDTA, 150 mM NaCl, 1% Triton X-100, 0.5% Protease Inhibitor Cocktail (Sigma, St. Luis, MO, USA), 2 mM Na_3_VO_4_, 10 mM NaF) using the TGF-beta 1 Quantikine ELISA Kit (R&D Systems, Minneapolis, MN, USA, Cat. No. DB100B) following the manufacturer’s user guide.

For human syndecan-1 ELISA, blood samples were collected and centrifuged for 10 min at 2400 rpm, and the plasma was transferred to a clean 1.5 mL tube. The amount of hSDC1 was quantified in the plasma using the CD138 (SDC1) ELISA Kit (Diaclone, Gen Probe, Besançone, France, Cat. No. 850.640.096) according to the manufacturer’s protocol.

Mouse syndecan-1 levels were determined from plasma (preparation described above) by indirect enzyme-linked immunosorbent assay. In brief, the wells of a microtiter ELISA plate (Sarstedt, Germany) were coated with 50 µL samples at 4 °C overnight. The plate was washed 5 times with phosphate-buffered saline (PBS) containing 0.05% v/v Tween-20, then the remaining protein-binding sites were blocked with 5% w/v non-fat dry milk (Bio-Rad) in PBS at 37 °C for 30 min. After the washing procedure, the plate was incubated with the mouse syndecan-1 antibody (SinoBiological Inc., Beijing, China, Cat. No.: 50641-RP02) dilution at 1:1500 in 3% *w*/*v* non-fat dry milk in PBS at 4 °C overnight. After another wash step, the plate was incubated with appropriate horseradish peroxidase (HRP)-conjugated secondary antibody (DakoCytomation, Glostrup, Denmark, #P0448, 1:2000) at 37 °C for 30 min. The last wash step was followed by incubation with 3,3′,5,5′-tetramethylbenzidine (TMB) solution (Sigma) for 15 min, and to stop the color reaction, 2 M H_2_SO_4_-soultion was performed.

Samples were evaluated from 4 mice per group. Each sample was performed in duplicate, and the mean values were used for statistical analysis. ELISA plates were read at 450 nm with a Labsystem Multiskan MS (Labsystems, Vantaa, Finland) plate reader.

### 2.4. Phospho-Receptor Tyrosine Kinase (pRTK) Array

Total proteins were extracted from frozen liver tissues. After homogenization in liquid nitrogen, 1 mL of lysis buffer was added to the samples (20 mM Tris pH 7.5, 2 mM EDTA, 150 mM NaCl, 1% Triton X-100, 0.5% Protease Inhibitor Cocktail (Sigma, St. Louis, MO, USA), 2 mM Na_3_VO_4_, 10 mM NaF). After incubation for 30 min on ice, samples were centrifuged at 15,000× *g* for 20 min. Supernatants were kept and protein concentrations were measured using the Bradford method. Pooled samples of five livers from the same experimental group were adjusted to 1.2 μg protein/μL lysate, and relative levels of receptor tyrosine kinase (RTK) phosphorylation were determined using the Proteome Profiler Array (R&D Systems, Minneapolis, MN, USA), according to the manufacturer’s instructions. Signals were developed by incubating the membrane in a SuperSignal West Pico Chemiluminescent Substrate Kit (Pierce/Thermo Scientific, Waltham, MA, USA), and visualized on a Kodak Image Station 4000MM Digital Imaging System.

### 2.5. Phosphorylation Antibody Array Analysis

The Cancer Signaling Phospho Antibody microarray (PCS248, Full Moon Biosystems, CA, USA) contained 248 antibodies, each of them in 6 replicates, printed on coated glass microscope slides along with multiple positive and negative controls. Protein extraction was performed according to the manufacturer’s protocol. The slides were scanned by SureScan Dx Microarray Scanner (Agilent Technologies, Santa Clara, CA, USA) and the density of the dots was quantified using free ImageJ (Version 1.50b, US National Institute of Health, Bethesda, MD, USA) software. The fluorescence intensity of each array spot was quantified, and mean values were used for statistical analysis.

### 2.6. Western Blot

Total protein of 30 μg amounts were mixed with loading buffer containing β-mercaptoethanol and incubated at 99 °C for 5 min. Denatured samples were loaded onto a 10% polyacrylamide gel and run for 30 min at 200 V on a Mini Protean vertical electrophoresis equipment (Bio-Rad, Hercules, CA, USA). Proteins were transferred to PVDF membrane (Millipore, Billerica, MA, USA) by blotting for 1.5 h at 100 V. Ponceau staining was applied to determine blotting efficiency. Membranes were blocked with either 3% w/v non-fat dry milk (Bio-Rad) or 5% *w*/*v* bovine serum albumin in TBS for 1 h and incubated with the primary antibodies at 4 °C for 16 h. Ponceau staining served as a loading control. Membranes were washed 5 times with TBS containing 0.05% *v*/*v* Tween-20 and incubated with appropriate secondary antibodies for 1 h. Signals were detected by SuperSignal West Pico Chemiluminescent Substrate Kit (Pierce/Thermo Scientific) and visualized on a Kodak Image Station 4000MM Digital Imaging System. Western blot analyses were performed 3 independent times, running the samples in duplicates. The density of the bands was measured by the software provided with Kodak Image Station. For antibody specifications and dilutions, see Table S1.

### 2.7. Immunohistochemistry

FFPE mouse liver sections were stained with HE for histopathological evaluation. Slides were immunostained using the Novolink Polymer Detection System (Peroxidase/DAB+, Rabbit, Novocastra Laboratories, Newcastle, UK). Endogenous peroxidase was inactivated by the addition of 10% H_2_O_2_ dissolved in methanol for 20 min. After antigen retrieval at 100 °C in Tris-EDTA buffer (10 mM Tris; 1 mM EDTA; 0.05% Tween-20; pH 9; 3 min), nonspecific binding was blocked for 10 min using Novocastra™ Protein Block. Primary antibodies (Table S1) were applied overnight at 4 °C, followed by either the Novolink Polymer for 30 min or appropriate secondary antibody-conjugated HRP (Table S1) for 1 h. Signals were visualized using 3,3-diaminobenzidine tetrahydrochloride substrate chromogen solution (Dako, Glostrup, Denmark), and counterstained with hematoxylin. Stained slides were scanned by a high-resolution bright field slide scanner (Pannoramic P1000, 3DHistech Ltd., Budapest, Hungary).

### 2.8. Proteomics, Bioinformatics

Unless otherwise stated, reagents and consumables were from Sigma-Aldrich (Sigma-Aldrich Gmbh., Budapest, Hungary).

#### 2.8.1. Surface Digestion of FFPE Tissues

FFPE tissue in 10 µm sections from WT and hSDC1^+/+^ CTL mice at month 6, as well as from WT and hSDC1^+/+^ DEN mice at months 3, 6, and 9, and hSDC1^+/+^ DEN mice at month 11, were prepared for liquid chromatography–mass spectrometry (LC–MS). Tissue dewaxing and antigen retrieval of the slides were performed as described earlier [19], and tissue areas corresponding to normal tissue, foci, and/or tumors were digested using trypsin on the tissue surface as reported before [20]. Briefly, for protein denaturation and reduction, 5 µL of a solution containing 0.1% RapiGest SF (Waters, Milford, MA, USA), 5 mM dithiothreitol, and 10% glycerol was added, and slides were incubated in a humidified box at 55 °C for 20 min. Next, 5 µL of a solution containing 25 mM ammonium bicarbonate (AmBic), 10 mM iodoacetamide, and 10% glycerol were added, and tissues were incubated at room temperature in the dark for 20 min. Subsequently, 5 µL of Trypsin/Lys-C mix (Promega, Madison, WI, USA) enzyme solution (50 ng/µL trypsin/Lys-C mix in 50 mM AmBic and 10% glycerol) and 5 µL of Trypsin (Promega, Madison, WI, USA) enzyme solution (200 ng/µL trypsin in 50 mM AmBic and 10% glycerol) were added in two and three cycles, respectively. In each proteolysis cycle, tissues were incubated in a humidified box for 40 min at 37 °C. After digestion, peptides were extracted from the tissue with 4 × 5 µL 10% acetic acid. Finally, samples were purified using Pierce C18 spin columns (Thermo Fisher Scientific, Waltham, MA, USA) according to the manufacturer’s protocol.

#### 2.8.2. Chromatography and Mass Spectrometry

Samples were dissolved in 12 µL solvent (98% water, 2% acetonitrile, and 0.1% formic acid), out of which 2.5 µL were injected into the nanoLC-MS/MS system consisting of a Dionex Ultimate 3000 RSLC nanoLC (Dionex, Sunnyvale, CA, USA) coupled to Bruker Maxis II Q-TOF apparatus (Bruker Daltonik GmbH, Bremen, Germany). Peptides were trapped on an Acclaim PepMap100 C18 (5 µm, 100 µm × 20 mm, Thermo Fisher Scientific, Waltham, MA) column and then separated on an Acquity M-Class BEH130 C18 analytical column (1.7 µm, 75 µm × 250 mm Waters, Milford, MA) using a gradient ranging from 4% to 50% eluent B in 120 min. Solvent A was water + 0.1% formic acid; Solvent B was acetonitrile + 0.1% formic acid. Spectra were collected using a fixed cycle time of 2.5 s and the following scan speeds: MS spectra at 3 Hz, while collision-induced dissociation (CID) was performed on multiply charged precursors at 16 Hz for abundant ones, and at 4 Hz for low abundance ones. Internal calibration was performed at the beginning of every measurement by sodium formate clusters and data were automatically recalibrated using Compass Data Analysis 4.3 (Bruker Daltonik GmbH, Bremen, Germany).

#### 2.8.3. Protein Identification and Label-Free Quantitation

Database search was performed by ProteinScape 3.0 (Bruker Daltonik GmbH). Proteins were identified by searching against the mouse Swissprot database (2015_08) using the Mascot search engine version 2.5.1 (Matrix Science, London, UK). During the Mascot search, the following search parameters were set: trypsin enzyme, 7 ppm precursor mass tolerance, 0.05 Da fragment mass tolerance, max. 2 missed cleavages, carbamidomethylation of cysteines as a fixed modification, deamidation (NQ), and oxidation (M) as variable modifications. Only those proteins were accepted that were identified with a minimum of two unique peptides and 1% false discovery rate (FDR). MaxQuant [21] software version 1.5.3.30 was used for label-free quantitation with the database created from the proteins formerly identified with ProteinScape using the default settings of the program.

#### 2.8.4. Protein Interaction Analysis

Protein interaction analysis was carried out using the “Search Tool for Recurring Instances of Neighboring Genes” (STRING) [22] webserver. Proteomics data were analyzed in two distinct datasets. First, changes in lipid metabolism 3 months after DEN treatment in both WT and hSDC1^+/+^ were mapped, and proteins that were upregulated at least by a factor of 3.0 (*p* < 0.05) in the DEN-exposed samples were selected for interaction analysis. Secondly, changes related to DEN treatment in both WT and hSDC1^+/+^ were identified in the full proteome at months 3, 6, and 9. Here, proteins that were upregulated or downregulated at least by a factor of 2.5 in each sample at the given time point were selected for network analysis. A full network was built with high interaction confidence (interaction score larger than 0.7), and disconnected nodes were hidden. The sources used for the evidence of interaction were the following: text mining (yellow), experiments (magenta), data bases (light blue), co-expression (dark grey), and neighborhood (light green). Proteomics measurement data have been submitted to the MassIVE repository under the submission number MSV000086679.

### 2.9. Statistical Analysis

Data were analyzed using GraphPad Prism v6.01 (GraphPad Software, La Jolla, CA, USA) and Microsoft Excel v.2016 (Microsoft Corp., Redmond, WA, USA).

In the case of Western blot, data from hSDC1^+/+^ CTL were normalized to WT CTL, and data from hSDC1^+/+^ DEN were normalized to WT DEN.

Data were analyzed by unpaired Student’s *t*-test in the case of Western blot, pRTK array, phosphorylation antibody array and MS, and by one-way ANOVA in the case of ELISAs. Significance levels were selected as * *p* < 0.05; ** *p* < 0.01; and *** *p* < 0.001.

## 3. Results

### 3.1. The Development of DEN-Induced Liver Tumors Was Delayed in hSDC1^+/+^ Mice

**Figure 2:**
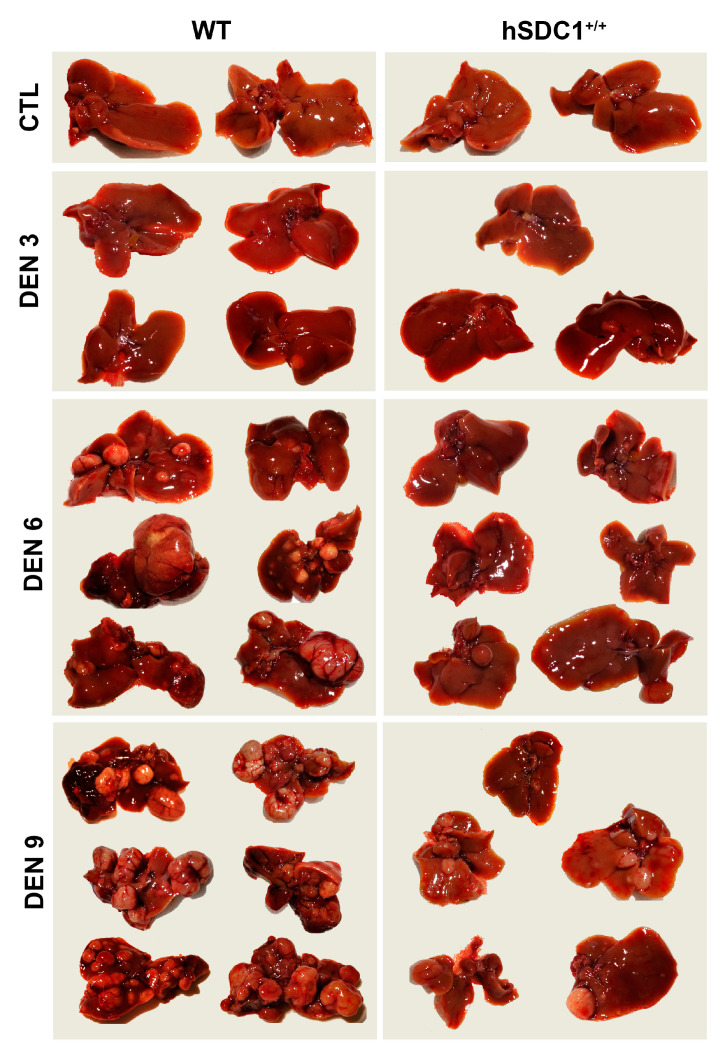
Diethylnitrosamine (DEN)-induced cancer development is delayed in hSDC1^+/+^ livers. Wild-type (WT) livers displayed several tumor nodules six months after DEN exposure, whereas only a single preneoplastic nodule was detected in one of the hSDC1^+/+^ livers. At 9 months, the livers of WT animals were largely occupied by cancer nodules and they started to die at this time. A comparable number of cancer nodules developed in hSDC1^+/+^ livers only by 11 months.

**Figure 3:**
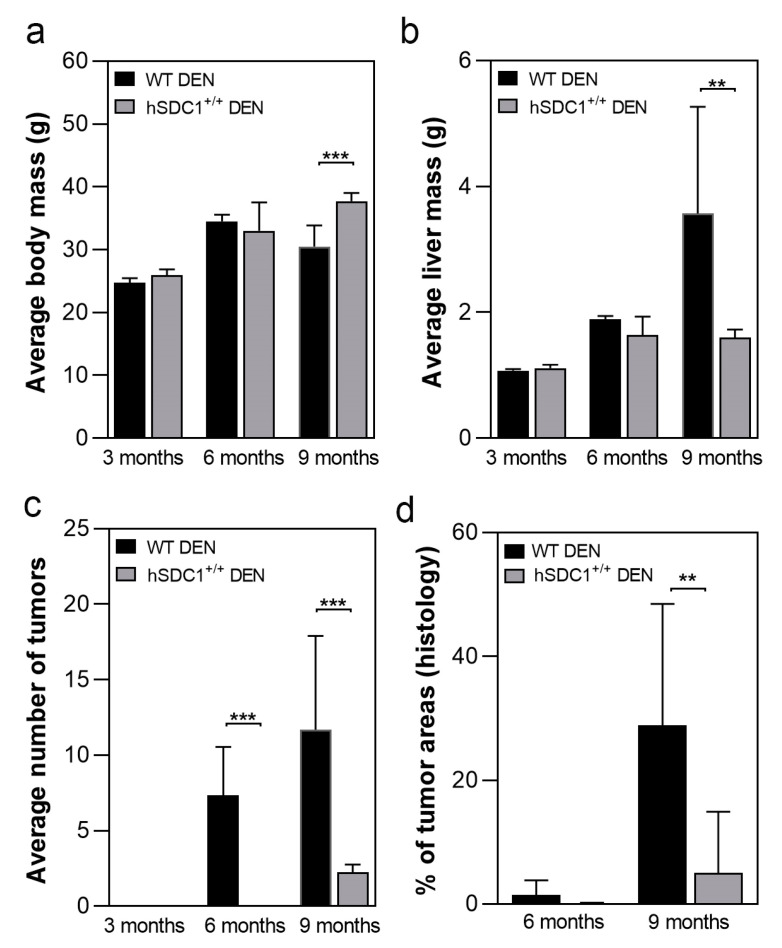
Macroscopic and histologic outcomes of DEN-induced carcinogenesis in WT and hSDC1^+/+^ mice. (**a**) Comparison of body mass of WT and hSDC1^+/+^ mice showed a difference only at 9 months, indicating weight loss of WT mice due to tumorous wasting. (**b**) At 9 months, significantly increased liver mass was measured in WT livers compared to hSDC1^+/+^, reflecting a large number of cancer nodules. (**c**) No macroscopically visible cancer was found in hSDC1^+/+^ livers at 6 months. At 9 months, hSDC1 livers contained, on average, 3–4-fold fewer tumor nodules compared to WT. (**d**) Histological examination revealed a few small tumor nodules at 6 months in hSDC1^+/+^ livers. The area occupied by tumors increased to 30% in WT but only 10% in hSDC1^+/+^ by month 9. Data points represent the mean ± standard deviation (SD), *n* of hSDC1^+/+^ DEN = 22, *n* of WT DEN = 14, ** *p* < 0.01; *** *p* < 0.001.

**Figure 4:**
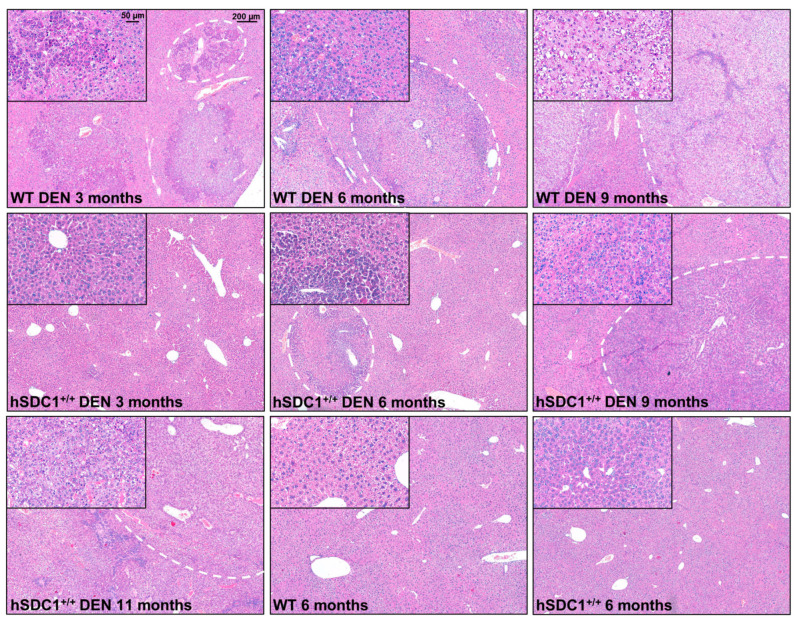
Liver histology of WT and hSDC1^+/+^ mice. The first row demonstrates cancer progression in WT DEN-treated livers. Preneoplastic foci already appeared at month 3, and their number increased by month 6 along with the emergence of overtly cancerous nodules. At month 9, WT livers were almost completely obliterated by cancer. The second row shows hSDC1^+/+^ livers at the same time points. No foci were seen at month 3. The first foci appeared at month 6; these grew further by month 9, and a few small neoplastic nodules developed. The left panel in the third row shows a representative DEN-treated hSDC1^+/+^ liver at month 11 with a high number of cancer nodules. Both WT and hSDC1^+/+^ were similar in histology with round-shaped polymorphic nuclei, clear cytoplasm, and frequent cell divisions. Untreated WT and hSDC1^+/+^ livers were essentially indistinguishable by histology. No sign of liver fibrosis or cirrhosis was detected. Representative images are at 50× (main pictures) and 200× (insets) magnification with scale bars of 200 μm and 50 μm, respectively. Representative scale bars are inserted into the first pictures.

A total of 125 animals, 68 males and 59 females, were enrolled in the carcinogenesis experiment (for group sizes in each experimental arm see Table 2). The development of tumors was followed up every third month after DEN exposure (months 3, 6, and 9). Due to the delayed development of tumors, the experiment was extended until month 11 for hSDC1^+/+^ mice. Age-matched control livers were taken in parallel with DEN-exposed livers. At each time point, 2–6 animals were sacrificed from each group. Tumorous nodules developed by month 6 and occupied the whole liver by month 9 in DEN-exposed WT animals; because no further propagation of cancer was expected and the animals started to die spontaneously, this experimental arm was terminated at month 9. As a contrast, at month 9, the number of tumors in hSDC1^+/+^ animals was still low, thus they were followed up until month 11 (Figure 2). Development of tumors was not accompanied by liver cirrhosis, which is a typical feature of DEN carcinogenesis. At month 9, the body mass of DEN-exposed WT animals was significantly lower compared to hSDC1^+/+^ because of severe wasting (Figure 3a), whereas their liver mass was significantly higher owing to high tumor burden (Figure 3b). Except for a few small preneoplastic foci, no detectable tumors were found at month 6, and on average fewer than three foci were identified at month 9 in hSDC1^+/+^ livers (Figure 3c), where sizable cancer nodules developed in larger numbers only by month 11 (Figure 4). Histological quantification of the areas occupied by cancer confirmed the macroscopic results (Figure 3d).

Hematoxylin–eosin staining of control livers showed typical liver histology. Untreated hSDC1^+/+^ livers displayed very similar morphology, except for a modest accumulation of desmin-positive perisinusoidal cells (Figure S1). Three months after DEN exposure, several preneoplastic foci were already seen in WT livers, whereas the histology of hSDC1^+/+^ livers was unchanged. Six months after DEN exposure, histologically overt HCC nodules developed in WT livers, while only a few foci could be found in hSDC1^+/+^ livers. By month 9, wild-type DEN-exposed livers were largely obliterated by cancer tissue; at the same time, scattered preneoplastic foci and no more than three suspected cancer areas per liver were detected in hSDC1^+/+^. Only hSDC1^+/+^ animals survived until month 11, at which time they were already bearing larger cancer nodules very similar to those developed in WT DEN-exposed livers. Their histology was dominated by tumor cells with clear, lightly basophilic cytoplasm and round-shaped polymorphic nuclei, and a considerable number of cell divisions (Figure 4).

### 3.2. Expression of Mouse and Human Syndecan-1 in the Livers of WT and hSDC1^+/+^ Mice

**Figure 5:**
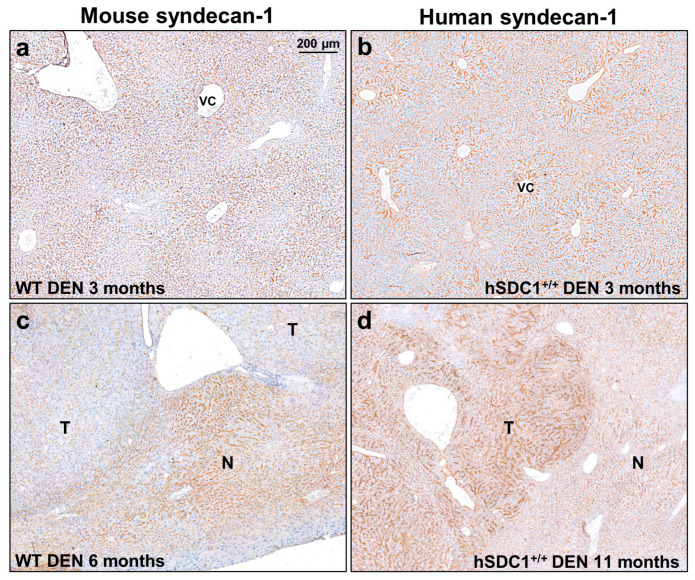
The immunolocalization of mouse and human syndecan-1 in WT and hSDC1^+/+^ livers. (**a**,**b**) Three months following DEN exposure, mouse syndecan-1 (mSDC1) as well as hSDC1 were concentrated around the central veins, with decreasing intensity toward the portal area. hSDC1 was more abundantly expressed compared to endogenous mSDC1. (**c**) At month 6, the expression of mSdc1 decreased in the tumorous areas compared to the normal parenchyma, in contrast with the hSDC1^+/+^ livers (**d**) where cancer nodules (shown at month 11) displayed upregulation of hSCD1. Representative images are at 50× magnification; scale bar: 200 μm. T, tumorous area; N, normal tissue; VC, vena centralis.

In untreated WT animals, mouse syndecan-1 (mSDC1) was localized to the pericentral region of liver lobules, and this pattern was maintained in the focus-free areas of DEN-treated WT livers at month 3 (Figure 5a). Interestingly, decreased intensity of mSDC1 was often observed in preneoplastic foci and tumors at month 6 after DEN exposure (Figure 5c).

The localization of hSDC1 in DEN-treated hSDC1^+/+^ mice at month 3 was very similar to that of mouse syndecan-1 in WT, although the staining was more intensive (Figure 5b). However, it must be emphasized that hSDC1 is driven by the albumin promoter while mSDC1 is driven by the endogenous SDC1 promoter. Thus, their transcription regulation does not occur in parallel. Of note, hSDC1^+/+^ mice expressed mSDC1 as well (Figure S2). In contrast with the tumors developed in WT, hSDC1^+/+^ tumors exhibited elevated expression of hSDC1 (Figure 5d).

### 3.3. Shedding of Mouse and Human Syndecan-1

**Figure 6:**
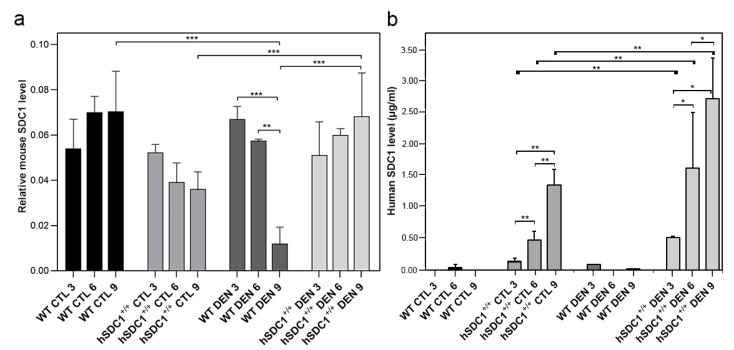
Shedding of mSDC1 and hSDC1 in control and DEN-exposed livers of WT vs. hSDC1^+/+^ mice. (**a**) The amount of mSDC1 released from WT control livers remained largely stable throughout the experimental period. Following DEN exposure, the shedding of mSDC1 became significantly downregulated in WT livers by month 9. In untreated hSDC1^+/+^ mice, the amount of shed mSDC1 and hSDC1 changed in opposite directions. In control mice, (**a**) shedding of mSDC1 decreased, (**b**) whereas shedding of hSDC1 increased. Shedding of both mSDC1 and hSDC1 increased over time in DEN-exposed hSDC1^+/+^ mice. Bars show mean ± SD, *n* = 3; * *p* < 0.05; ** *p* < 0.01; *** *p* < 0.001.

Shedding of mSDC1 was essentially invariant in WT and hSDC1^+/+^ controls throughout the nine-month follow-up period, while it dropped significantly by month 9 in DEN-exposed WT livers (*p* < 0.05 and 0.001, respectively). No significant changes were observed in the shedding of mSDC1 in DEN-exposed hSDC1^+/+^ livers, but a significant difference was observed between hSDC1^+/+^ CTL and hSDC1^+/+^ DEN mSDC1 expression at month 9 (Figure 6a). Conversely, the shedding of hSDC1 gradually increased in both control and DEN-exposed hSDC1^+/+^ livers, with the increase being significantly higher in the latter (*p* < 0.01) (Figure 6b).

### 3.4. pRTK Array Indicates Downregulation of Receptor Activation in DEN-Treated hSDC1^+/+^ Mice

**Figure 7:**
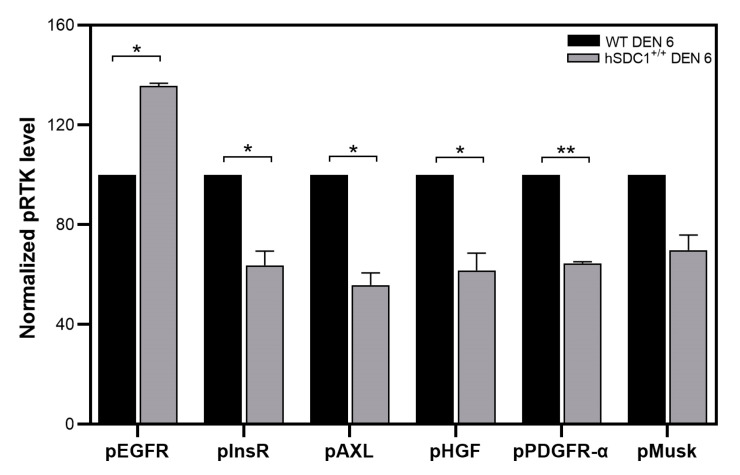
Receptor tyrosine kinase activation in WT and hSDC1^+/+^ livers 6 months after DEN exposure. Except for pMusk, significant inhibition of all receptors was detected in hSDC1^+/+^ livers. Heparan sulfate (HS) binding epidermal growth factor (EGF) establishes heterodimer with shed hSDC1 binding together to EGFR and facilitate the activation of the receptor. Data points represent the mean ± SD, *n* = 3; * *p* < 0.05; ** *p* < 0.01.

A pRTK array revealed significantly lower activating phosphorylation of insulin (InsR), platelet-derived growth factor (PDGF), hepatocyte growth factor (HGF) and Axl receptors, but significantly higher activation of epidermal growth factor receptor (EGFR) in DEN-treated hSDC1^+/+^ mice compared to that of WT at month 6 (Figure 7 and Figure S7).

### 3.5. Different Early Phase Response to DEN Exposure in the Lipid Metabolism of WT vs. hSDC^+/+^ Mice

**Figure 8:**
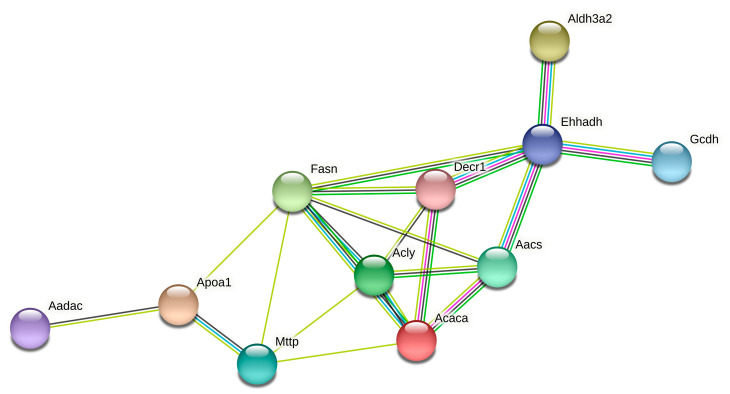
Connectedness graph of 11 proteins overexpressed in WT DEN livers. Edge weights represent the strength of co-regulation.

**Figure 9:**
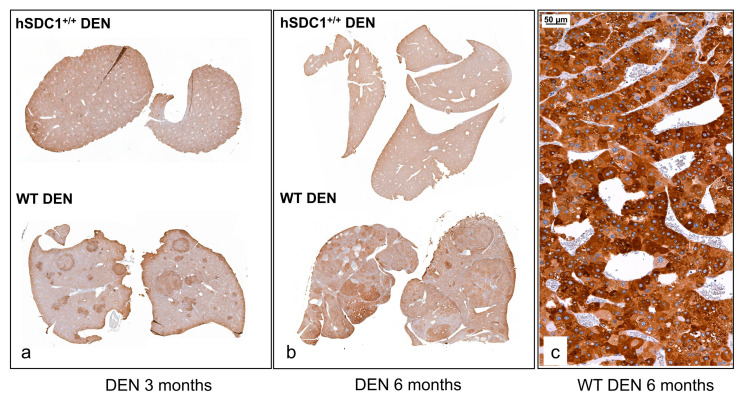
Fasn immunostaining in WT DEN and hSDC1^+/+^ DEN livers. (**a**,**b**) A low amount of homogenously distributed cytoplasmic reaction was seen in hSDC1^+/+^ DEN livers at month 3 and month 6. In WT livers, elevated immunostaining was detected in the preneoplastic foci at month 3 as well as in tumors at month 6. © At high magnification, intense cytoplasmic Fasn immunostaining was observed in the cytoplasm of cancer cells in WT DEN tumors. Representative image at 200× magnification, scale bar: 50 μm.

Quantitative mass spectrometry (qMS) analysis of WT DEN and hSDC1^+/+^ DEN livers at month 3 revealed more than 10-fold higher expression of Ehhadh, Fasn, and Acly proteins, all involved in lipid metabolism, in the foci developed in WT DEN livers compared to hSDC1^+/+^ DEN liver parenchyma (no foci were present in hSDC1^+/+^ DEN at this early time point). STRING analysis detected eight other proteins also implicated in fat metabolism with 3.1–7.6-fold higher expression in the foci of WT livers (Table 3 and Figure 8). Consistently, immunohistochemistry of WT DEN livers showed high Fasn positivity in preneoplastic foci at month 3 as well as in overt cancers at month 6, whereas no Fasn overexpression was detected in hSDC1^+/+^ DEN livers at either time point (Figure 9). When comparing whole tissue homogenates by qMS, the difference between WT DEN and hSDC1^+/+^ DEN in Fasn protein levels was only two-fold at month 3, consistent with the notion that whole tissue homogenates of WT DEN livers contained non-overexpressing normal parenchyma admixed with overexpressing foci and/or tumors (Figure S3).

### 3.6. TGF-β1 Expression in Control and DEN-Exposed Livers

Three months after the start of the experiment, no major differences in TGF-β1 expression were seen across the groups. TGF-β1 levels dropped by month 6 and returned to initial levels by month 9 in WT CTL; the relevance of these changes remains unclear. At month 6, TGF-β1 levels were about two-fold higher in hSDC1^+/+^ DEN mice compared to WT DEN; however, by month 9, this relationship was inversed because TGF-β1 was markedly upregulated in WT DEN while it remained invariant in hSDC1^+/+^ DEN, resulting in a three-fold difference in favor of WT DEN (Figure S4).

### 3.7. Downstream Signaling Effects of the Overexpression of hSDC1

#### 3.7.1. Western Blots Indicate Attenuated Pro-Proliferatory Signals in hSDC1^+/+^ DEN

**Figure 10:**
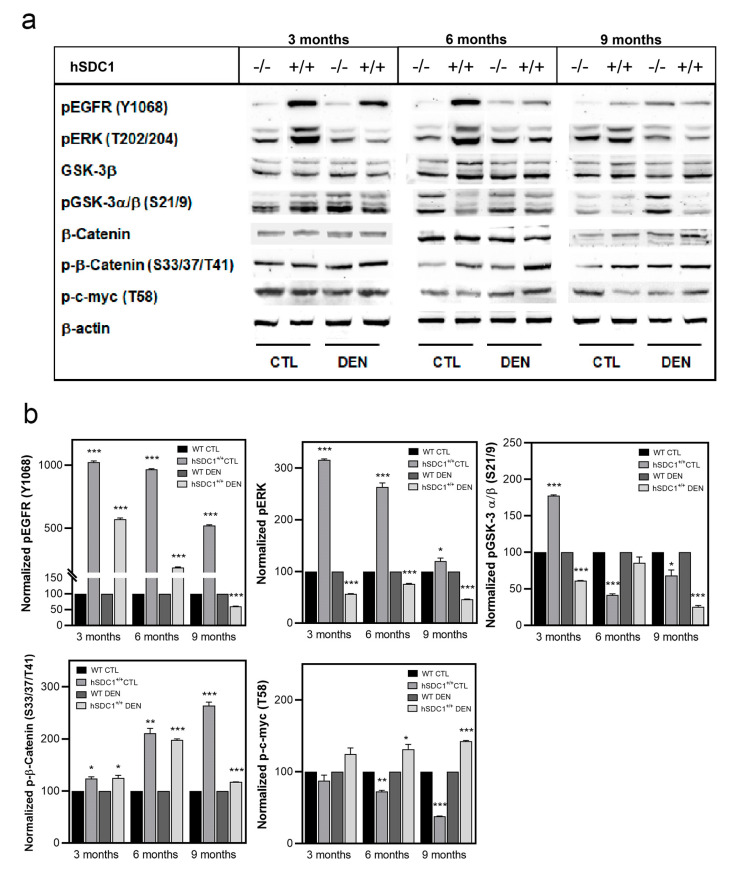
Western blot analysis of selected components of EGFR and β-catenin signaling. (**a**) Immunoblots and (**b**) corresponding densitometry graphs. Data points represent mean ± SD, *n* = 3; * *p* < 0.05; ** *p* < 0.01; *** *p* < 0.001.

EGFR and β-catenin signaling, the mTOR pathway, and the cell cycle regulators p53, p21, and p27 were analyzed on immunoblots. Activating Y1068 phosphorylation of EGFR remained low throughout in WT livers, both in CTL and DEN, whereas high EGFR activation was observed in hSDC1^+/+^ CTL at months 3 and 6, and in hSDC1^+/+^ DEN at month 3. pERK202/204 also displayed strong activation in hSDC1^+/+^ CTL, but not in hSDC1^+/+^ DEN, where the lowest pERK202/204 levels were seen throughout the experiment. While no significant differences were detected in the overall expression of GSK-3β or β-catenin, inhibitory S21/9 phosphorylation of GSKα/β was significantly decreased in hSDC1^+/+^ CTL and DEN livers at months 6 and 9, together with increased inhibitory phosphorylation of β-catenin (S33/37/T41) and c-myc (T58) in hSDC1^+/+^ DEN. Phospho-c-myc (T58) levels changed oppositely in hSDC1^+/+^ CTL, showing a decrease over time (Figure 10 and Figure S8).

**Figure 11:**
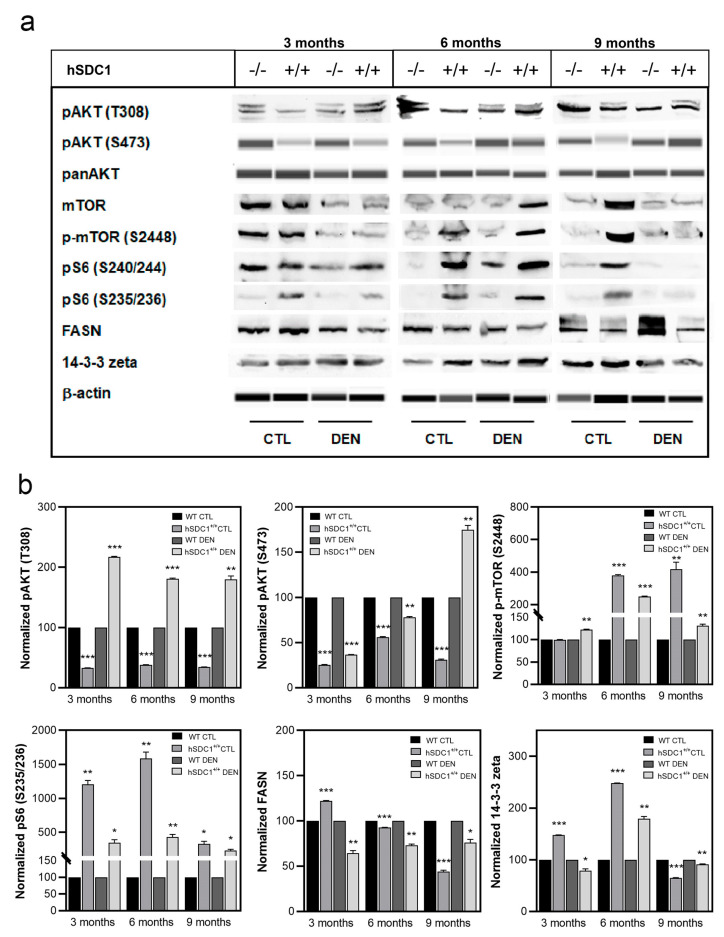
Western blot analysis of selected components of the Akt/mTOR pathway. (**a**) Immunoblots and (**b**) corresponding densitometry graphs. Data points represent mean ± SD, *n* = 3; * *p* < 0.05; ** *p* < 0.01; *** *p* < 0.001.

Changes in the activity of mTOR pathway verified a protective role of hSDC1 overexpression against carcinogenesis. Phosphorylation of Akt on T308 was significantly lower in hSDC1^+/+^ CTL compared to WT CTL, but markedly elevated in hSDC1^+/+^ DEN compared to WT DEN at all time points. pAKT (S473), on the other hand, was diminished in all hSDC1^+/+^ livers, both CTL and DEN, except for hSDC1^+/+^ DEN at month 9. Meanwhile, inactivating phosphorylation of mTOR (S2448) was strikingly upregulated in hSDC1^+/+^ livers, especially in CTL at months 6 and 9, and DEN at month 6. In spite of this suppression of mTOR in hSDC1^+/+^ livers, activating S235/236 phosphorylation of the ribosomal S6 subunit was consistently higher in all hSDC1^+/+^ samples, both in CTL and DEN, compared to the respective WT groups, especially at months 3 and 6 in hSDC1^+/+^ CTL. In whole tissue extracts used for immunoblotting, differences in Fasn expression were not as readily apparent by immunostaining or quantitative mass spectrometry; nevertheless, Fasn levels in WT DEN exceeded the levels measured in hSDC1^+/+^ DEN at months 3 and 6. Inactivating phosphorylation of 14-3-3-zeta was elevated with respect to WT in hSDC1^+/+^ CTL livers at months 6 and 9, as well as in hSDC1^+/+^ DEN samples at month 6 (Figure 11 and Figures S9 and S10).

**Figure 12:**
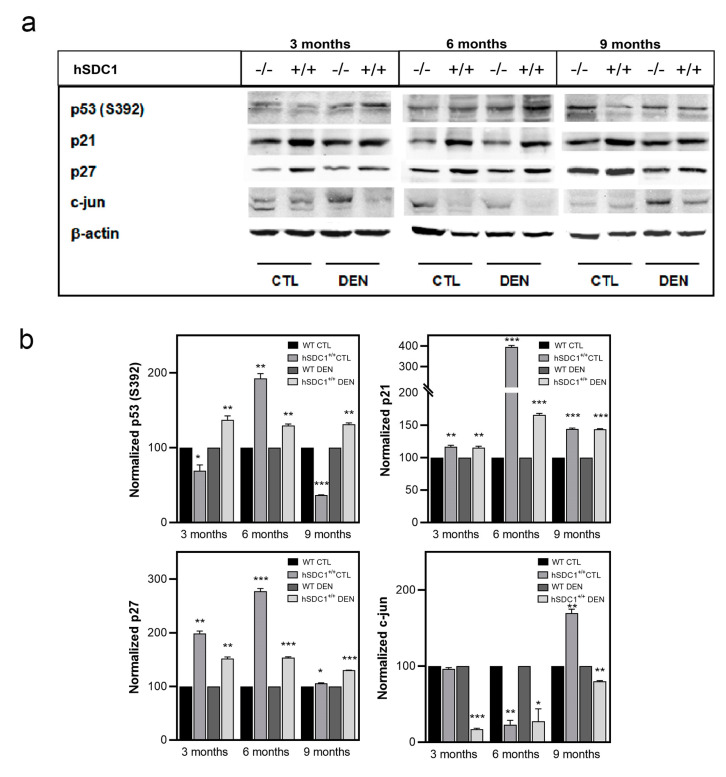
Western blot analysis of the cell cycle regulators p53, p21, p27, and c-jun. (**a**) Immunoblots and (**b**) corresponding densitometry graphs. Data points represent mean ± SD, *n* = 3; * *p* < 0.05; ** *p* < 0.01; *** *p* < 0.001.

The tumor suppressor p53 was hyperactivated by S392 phosphorylation in hSDC1^+/+^ DEN compared to WT DEN throughout, further suggesting an oncoprotective effect of hSDC1 overexpression. The cyclin-dependent kinase inhibitors p21 and p27 were similarly upregulated at all time points in hSDC1^+/+^ DEN and, albeit to a lesser extent, also in hSDC1^+/+^ CTL compared to the WT counterparts. Except for hSDC1^+/+^ CTL at month 9, the expression of c-jun was oppositely regulated in the same groups, consistent with attenuated cell cycling (Figure 12 and Figure S11).

#### 3.7.2. Immunohistochemistry Reveals Further Differences between WT DEN and hSDC1^+/+^ DEN Livers at Month 3

**Figure 13:**
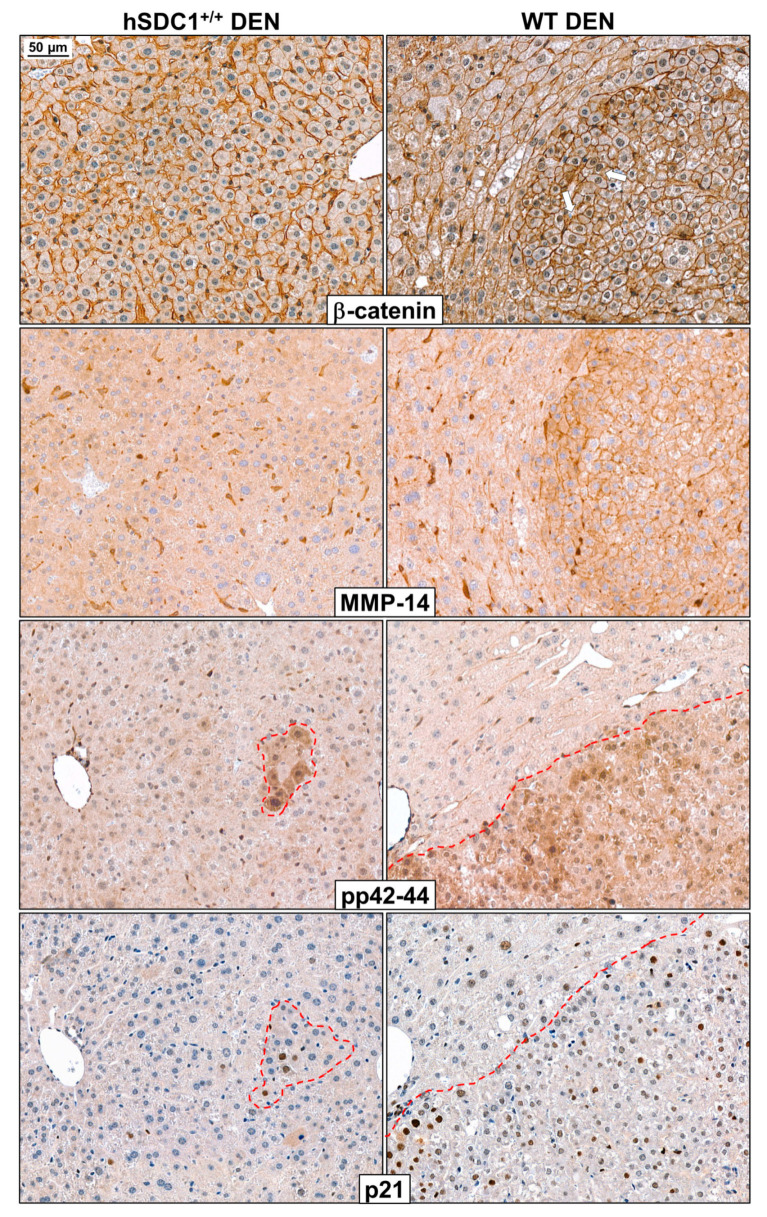
Immunostaining of β-catenin, MMP-14 (MT-MMP1), pp42-44 (pERK1/2), and p21 in WT DEN and hSDC1^+/+^ DEN livers at month 3. In hSDC1^+/+^ DEN livers with retained structure and devoid of premalignant foci, β-catenin was localized exclusively to the cell surface of hepatocytes. In the preneoplastic foci of WT DEN livers, polymorphic cells already displayed nuclear β-catenin positivity. In the same foci, strong immunostaining of MMP14, a metalloprotease known to be implicated in SDC1 shedding, was seen on cell surfaces, whereas only perisinusoidal cells but no normal hepatocytes expressed MMP14 in hSDC1^+/+^ DEN livers. In WT DEN livers, preneoplastic foci were extensively marked by pp42-44 positivity, whereas only small islets of cells displayed high pERK1/2 in hSDC1^+/+^ DEN. Areas with high pERK1/2 exhibited concomitant activation of the cyclin-dependent kinase inhibitor p21. White arrows show the nuclear β-catenin in the tumor area. Images are at 200× magnification, scale bar: 50 μm.

Immunostaining of β-catenin, MMP-14 (MT-MMP1), pERK1/2 (pp42-44) and p21 highlighted further important differences between WT DEN and hSDC1^+/+^ DEN that were already evident as early as three months after DEN exposure (Figure 13). As mentioned before, unlike WT DEN livers, hSDC1^+/+^ DEN livers did not develop preneoplastic foci by this time. In WT DEN, β-catenin was detectable in the nuclei of cells in preneoplastic foci, whereas only normal cell surface reaction was seen in hSDC1^+/+^ DEN. The same foci in WT DEN abundantly expressed MT-MMP1, a protease potentially implicated in SDC1 shedding, on their cell surface, whereas MT-MMP1 only resided in the perisinusoidal cells in hSDC1^+/+^ DEN samples. Preneoplastic foci in WT DEN were also characterized by intensive cytoplasmic pERK1/2 (pp42-44) staining, an alteration so specific that it could be regarded as an early marker of transformation. pERK1/2-high cells formed only small, scattered groups in hSDC1^+/+^ DEN livers. p21 immunostaining on adjacent serial sections revealed that the same pERK1/2-high cells expressed high levels of this important cyclin-dependent kinase inhibitor.

#### 3.7.3. Phospho-Kinase Array at Month 6 Confirms Suppressed Cancer Progression in hSDC1^+/+^ DEN

**Figure 14:**
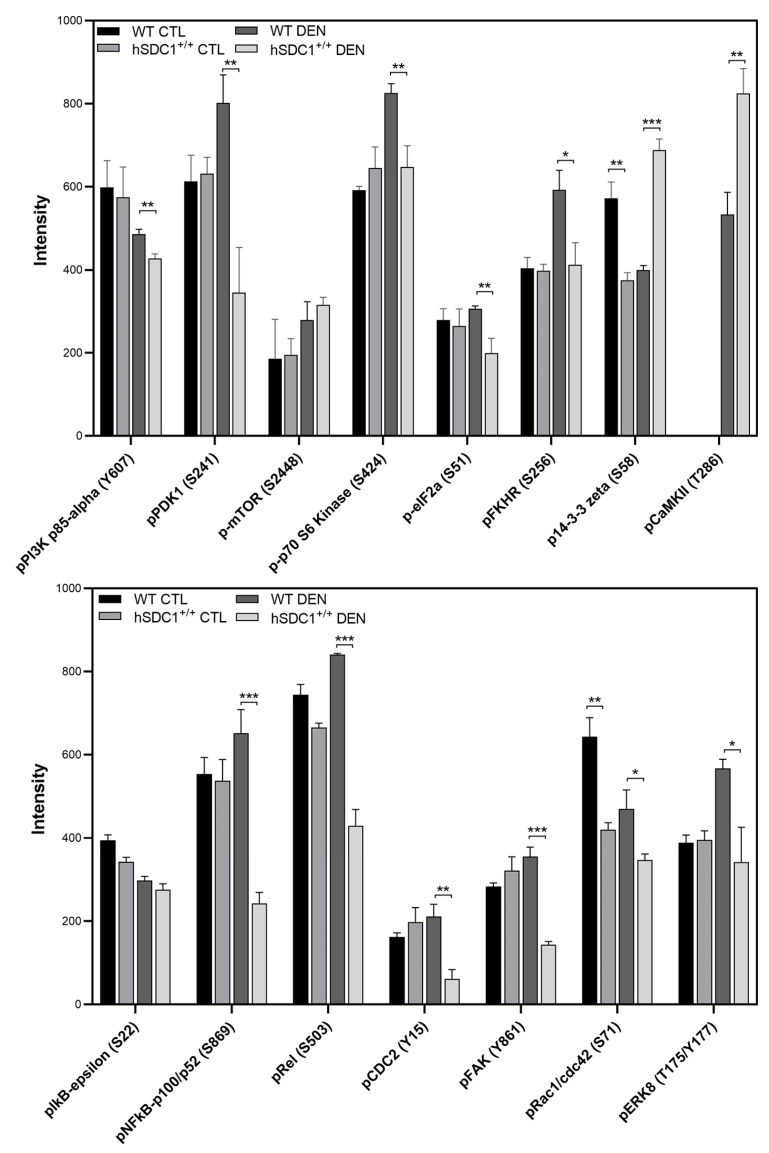
hSDC1-related alterations in cancer pathways revealed by the Full Moon Phospho Array. Data points represent the mean ± SD, *n* = 3; * *p* < 0.05; ** *p* < 0.01; *** *p* < 0.001.

An immunoarray of 269 antibodies was used to probe the phosphorylation status of cancer signaling pathways. Alterations in 15 phosphoproteins could be linked to the effect of hSDC1 overexpression in DEN-treated livers at month 6. Out of these, five components of the mTOR pathway—the PI3 kinase p85-alpha, PDK1, mTOR, p70 S6 kinase, and eIF2a—were significantly suppressed in hSDC1^+/+^ DEN samples compared to WT DEN. Additionally, a decrease in the activating phosphorylation of FAK at Y861 was seen, which may also attenuate the mTOR pathway. Further relevant and significant differences observed in hSDC1^+/+^ DEN compared to WT DEN included (i) decreased inhibitory phosphorylation of Foxo (FKHR)1, suggestive of its preserved potential to inhibit cell cycle progression; (ii) increased inhibitory phosphorylation of 14-3-3 zeta, capable of influencing several metabolic events and activating apoptosis; (iii) decreased phosphorylation of NF-κB p100/52 and Rel, associated with their lower transcription factor activity; (iv) downregulation of pCDC2 (pCDK1), also indicating inhibition of the entry to cell cycle; and (v) decreased phosphorylation of ras-related C3 botulinum toxin substrate 1 (RAC) that inhibits cancer development by decreased PAK1-mediated transactivation of β-catenin (Figure 14 and Figure S12).

### 3.8. Proteomics Analysis and STRING Interaction Networks

The proteomes of WT CTL, WT DEN, hSDC1^+/+^ CTL and hSDC1^+/+^ DEN livers were compared at months 3, 6, and 9. Proteins that were differentially expressed, i.e., that were uniquely present in only one group or exhibited 2.5-fold or greater difference between groups, were evaluated by STRING analysis. Differentially regulated proteins at month 11 are only tabulated (Tables S2 and S3) because no WT DEN animals were alive at this time point for comparison.

#### 3.8.1. Untreated (WT CTL and hSDC1^+/+^ CTL) Livers

In WT CTL livers, the relatively few proteins that were differentially regulated included ribosomal and proteasomal proteins, as well as proteins related to vesicular transport, clathrin-mediated endocytosis, xenobiotic metabolism, and detoxification. The ribosome group contained more 60S and 40S ribosome proteins in hSDC1^+/+^ CTL samples, and besides two proteasome proteins, mitochondrial electron transport was also represented by five proteins. A mixed cluster contained lipid metabolism-related proteins, proteins for G protein binding factor, and IGF binding factor, among others (Figure S5, Table S4).

#### 3.8.2. Alteration in DEN-Exposed Liver at Month 3

**Figure 15:**
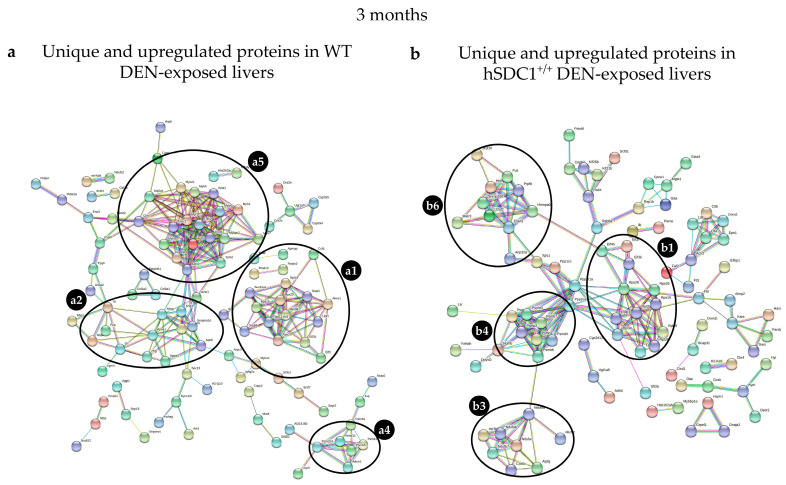
STRING analysis of (**a**) WT DEN and (**b**) hSDC1^+/+^ DEN liver proteomes at month 3. Labels: (**a1**) ribosomal proteins; (**a2**) apoproteins, serine proteases, and fetuin; (**a4**) proteasomal proteins; (**a5**) motor proteins differentially regulated in WT DEN; (**b1**) ribosomal proteins; (**b3**) mitochondrial respiratory chain proteins; (**b4**) proteasomal proteins; (**b6**) miscellaneous, including splicing related factors, RNA binding protein, nuclear RNP helicase, and nucleocytoplasmic transport molecules, differentially regulated in hSDC1^+/+^ DEN.

Three months after DEN exposure, the most abundant cluster in the WT DEN liver contained motor and motor-associated proteins such as myosins, tropomyosins, actins, and troponins. The ribosomal and proteasomal clusters contained fewer proteins, and different ones, compared to hSDC1^+/+^ DEN. Serine and other protease inhibitors formed a cluster together with high-density lipoprotein (HDL) components ApoA1, ApoB, and ApoA4 as well as plasma glycoprotein and fetuin. In hSDC1^+/+^ DEN samples, the ribosome and proteasome clusters were more strongly represented, and members of the mitochondrial electron transport were also present. A fourth cluster contained splicing-related factors, RNA binding protein, nuclear RNP helicase, and nucleocytoplasmic transport molecules (Figure 15, Table 4).

#### 3.8.3. Alterations in DEN-Exposed Livers at Month 6

In WT DEN livers, the motor protein cluster was limited to Acta and Myh4, and only five ribosomal proteins were differentially expressed. In the cluster that included HDL components, serpins, Apoa1 and its binding protein Apoe, Calu, and Pzp occupied dominant positions. Retinol binding proteins and galectin were also upregulated. In hSDC1^+/+^ DEN livers, additional upregulated ribosomal proteins included five Rpl and five Rps members and were joined by five eukaryotic translation initiation factors. Proteasomal and mitochondrial electron transport clusters were more heavily populated, too. Components differentially regulated in hSDC1^+/+^ DEN but not in WT DEN included proteins involved in pre-mRNA splicing, vesicular transport, and membrane trafficking (Figure S6, Table S5).

#### 3.8.4. Alterations in DEN-Exposed Livers at Month 9

**Figure 16:**
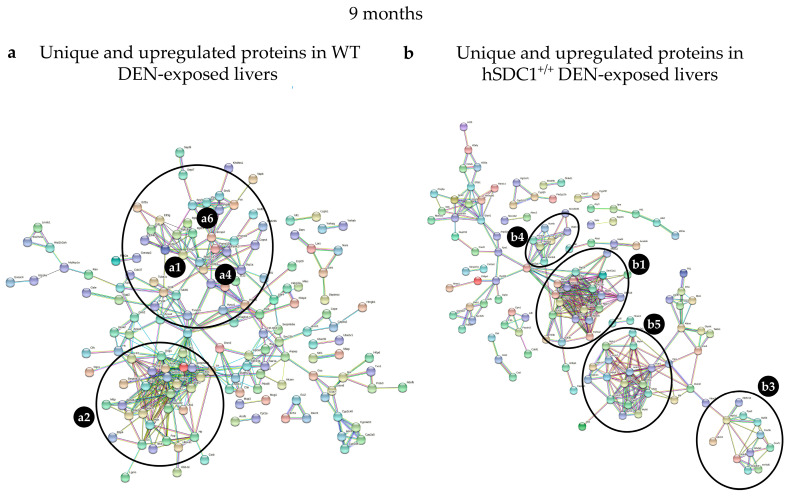
STRING analysis of (**a**) WT DEN and (**b**) hSDC1^+/+^ DEN liver proteomes at month 9. Labels: (**a1**) ribosomal proteins; (**a4**) proteasomal proteins; (**a6**) splicing factors (all forming a single cluster); and (**a2**) apoproteins and lipid metabolism proteins (forming a separate cluster) differentially regulated in WT DEN; (**b1**) ribosomal proteins; (**b3**) respiratory chain proteins; (**b4**) proteasomal proteins; and (**b5**) motor proteins differentially regulated in hSDC1^+/+^ DEN.

The STRING network of WT DEN liver at month 9 was clearly dominated by the cluster containing Apo proteins. An increased number of apoproteins including ApoA1, Apo4, ApoE, ApoC2, C3, and Apoh, was joined by Serpin a1b, a1c, and f2, as well as Pzp, Ola, and Mttp. Clusterin, a protein related to HCC, appeared along with a small number of ribosomal and proteasomal proteins and components of endoplasmic stress. In hSDC1^+/+^ DEN livers, the appearance of motor proteins was the first clear mark of cancer that was delayed by 6 months compared to WT DEN. Ribosomal proteins in the graph were similar in number but differed in identity from those detected at month 6, and the number of mitochondrial electron transfer proteins decreased (Figure 16, Table 5).

## 4. Discussion

### 4.1. Hepatocarcinogenesis

Our previous experiments indicated that SDC1, the major proteoglycan of the liver, is capable of inducing hepatocyte-like differentiation of hepatoma cell lines [16], and HS chains in the healthy human liver can interfere with DNA-binding transcription factors [23]. Together, these results suggested that the regulatory functions of SDC1 in the liver may be far more extensive than previously thought.

This model allowed us to assess the mechanism of the presumed protective role of SDC1 against the development of HCC and find an explanation for its beneficial action. In our model, hSDC1 expression was driven by the mouse albumin promoter and was thus exclusively restricted to the liver. It turned out that this model rather mimics the nonalcoholic steatohepatitis type cancer evolution. Hepatocarcinogenesis was followed up in transgenic and wild-type animals, and a six month delay in the formation of overt cancer was observed in hSDC1^+/+^ mice, confirming the assumption that SDC1 suppresses oncogenesis in the liver. Remarkably, all WT DEN animals died by the end of month 9, whereas macroscopic tumors appeared in large numbers only as late as month 11 in hSDC1^+/+^ DEN mice. After a six month delay, the cancer which developed in hSDC1^+/+^ DEN-exposed livers was very similar to that observed in wild-type livers. It seems that the genetic injury caused by DEN exposure cannot be repaired, and the protective effect of syndecan-1 is exhausted after a while. In spite of that, this result provided clear indication that the liver belongs to those organs where, unlike the breast or the pancreas [24,25,26], SDC1 exerts an oncoprotective role.

It has to be mentioned that overexpression of syndecan-1 induced certain changes in control livers without DEN exposure as well. However, these were rather beneficial, such as mTOR pathway inhibition, decreased Fasn concentration throughout the whole experimental period, or protecting against GSK-3β inhibitory phosphorylation, downregulation of c-jun, and upregulation of p21 and p27. All of these changes did not result in morphological alterations of the livers.

### 4.2. Changes in Syndecan-1 Shedding and Receptor Tyrosine Kinase Activity

hSDC1^+/+^ mice expressed both human and mouse syndecan-1, and despite a marked increase in hSDC1 shedding over time, abundant hSDC1 was retained on the cell surface in hSDC1^+/+^ DEN tumors. In contrast, in WT DEN mice the amount of mouse syndecan-1, both on the cell surface and shed in the plasma, gradually decreased over the nine months of observation. Syndecan-1 is known to establish interactions with high-affinity tyrosine kinase receptors; therefore, it looked conceivable that its accelerated shedding might influence kinase activity, and elevated shedding in hSDC1^+/+^ DEN may contribute to the removal of growth factors from the tumor cell surface and thereby attenuate the activation of cell surface receptors. Indeed, significant downregulation of insulin, Met, platelet-derived growth factor receptor (PDGFR), and AXL receptor activities was confirmed in the liver of hSDC1^+/+^ DEN animals at month 6 compared to WT DEN. This already hinted at changes in metabolic regulation, because at least two of these receptors are intimately involved in intermediary metabolism [27,28,29], which is now widely acknowledged to be involved in carcinogenesis [27]. The implication of syndecans in intermediary metabolism has never been a major focus in syndecan research, but at least one study of the effect of Sdc-4 polymorphism on glucose metabolism in flies has called attention to such potential of syndecans and looked for parallel events in whole-body energy metabolism in humans [29]. Here, consistent with our finding, the mTOR pathway and Foxo1 activity were identified as key mediators between syndecans and metabolism. An explanation for the high activation of EGFR is still lacking, although it was probably related to the interaction of heparin-binding EGF growth factor, with the HS chains of shed SDC1 and stimulation of EGFR by the EGF–HS complex [30].

### 4.3. Alterations in Lipid Metabolism

The proteomes of WT DEN and hSDC1^+/+^ DEN were first compared three months after DEN exposure. At this time point, hSDC1^+/+^ DEN livers displayed no sign of carcinogenesis, whereas WT DEN livers already contained numerous preneoplastic foci. Being aware of the role of SDC1 as lipoprotein receptors implicated in the clearance of LDL [11,31], as well as its role in adipocyte differentiation [32], we were anticipating differences between the groups in lipid metabolism. Indeed, dramatic enhancement was detected in WT DEN livers in proteins related to lipid metabolism. Fasn and Acly, critical components of lipid synthesis, were significantly upregulated in the preneoplastic foci of WT DEN, along with nine other components involved in lipid metabolism. Importantly, when early preneoplastic foci were first found at month 6 in hSDC1^+/+^ DEN livers, Fasn expression remained low, indicating a protective role of hSDC1 overexpression against this critical event of hepatocarcinogenesis. It is known from experimental models that increased lipogenesis is a characteristic feature of hepatocellular carcinomas, and Fasn and Acly are key actors in this process [33]. These proteins are also required for Akt- and c-Met-driven hepatocarcinogenesis [34]. Our mass spectrometry results were confirmed by immunohistochemistry, where strong Fasn positivity was detected in the preneoplastic foci and tumors of WT DEN livers. Lower Fasn expression remained a hallmark of hSDC1^+/+^ DEN at later time points of carcinogenesis, and Fasn was downregulated in hSDC1^+/+^ CTL livers as well.

Fasn activation promotes human cancer development, too, as was demonstrated in lung, pancreas, breast, ovarian, prostate, and colon cancers, which highlights the importance of lipogenesis in the development and progression of these tumors [35]. Inhibition of Fasn was shown to downregulate the β-catenin and mTOR signaling pathways [36], which is in good agreement with our findings.

### 4.4. Changes in the Levels of TGF-β1

TGF-β1 is oncoprotective in the early phase of hepatocarcinogenesis but promotes epithelial-to-mesenchymal transition (EMT) in the late phase of tumor formation [37]. SDC1 may inhibit the action of TGF-β1 either by binding and removing its activator thrombospondin or by directly sequestering TGF-β1 [17]. In our model, no significant differences in the amount of TGF-β1 were observed at month 3, but significant upregulation was found in hSDC1^+/+^ DEN livers at month 6, which may reflect the protective effect of TGF-β1. Consistent with literature data, marked upregulation of TGF-β1 was detected in WT DEN livers at month 9 when livers were already largely obliterated by tumors; in this context, the overexpression of TGF-β1 was more likely attributable to ongoing EMT [38].

### 4.5. Hepatocarcinogenesis Modified in hSDC1^+/+^ Mice is Characterized by the Downregulation of β-Catenin and mTOR Pathways

#### 4.5.1. Downregulation of the β-Catenin Pathway

Western blots revealed that in hSDC1^+/+^ DEN livers, the inactivating phosphorylation of GSK-3α/β at S21/9 decreased, resulting in increased phosphorylation and consecutive degradation of β-catenin. This finding received further corroboration from immunohistochemistry, where nuclear β-catenin positivity was detected only in WT DEN but not in hSDC1^+/+^ DEN livers. An additional mechanism possibly involved in the downregulation of β-catenin was the elevation of CaMKII in hSDC1^+/+^ DEN. CaMKII has been described to interfere with the heterodimer formation of T cell factor and β-catenin through the noncanonical pathway [39]. Decreased RAC phosphorylation in hSDC1^+/+^ could also hinder β-catenin activation through decreased PAK activation [39]. GSK3 activation resulted in inactivating myc phosphorylation on T58 in hSDC1^+/+^ tumors, which synergized with the above mechanisms in the suppression of the β-catenin pathway [40].

#### 4.5.2. Downregulation of the mTOR Pathway and Associated Changes in Intermediary Metabolism

The lack of Fasn upregulation in hSDC1^+/+^ DEN indicated the potential of overexpressed SDC1 to modulate tumor-associated changes in intermediary metabolism. Relatively low Fasn levels in hSDC1^+/+^ DEN were accompanied by decreased phosphorylation of PI3K 85-alpha, PDK1, and Akt (S481), while high Akt (S481) phosphorylation in WT CTL and DEN livers reflected high activity of the mTORC2 complex. mTOR, the central element of the mTORC1 complex, carried the inactivating Ser2448 phosphorylation in hSDC1^+/+^ samples, as confirmed by both Western blot and tyrosine kinase array. This inhibitory phosphorylation of mTOR is placed by pS6 kinase phosphorylated on T239, which acts as an executor in the feedback regulatory loop [41]. Phosphorylation of the same pS6 kinase at S424 is responsible for the activation of pS6 [42], a modification that initiates the biogenesis of the 40S ribosomal subunit. In hSDC1^+/+^ DEN tumors this phosphorylation was decreased; thus, pS6 had to be activated through an alternative mechanism. Theoretically, one option could be ERK1/2, because it was strongly upregulated in untreated hSDC1^+/+^ livers, and one of its downstream effectors, RSK1, is known to stimulate pS6 [43]. This, however, could not be the case in hSDC1^+/+^ DEN livers, where ERK1/2 activity was markedly suppressed at months 3 and 6, and pS6 kinase phosphorylation remained low at month 6. How pS6 could display high levels of phosphorylation in hSDC1^+/+^ DEN livers remains unclear until its activating factors are identified. In contrast with hSDC1^+/+^ samples, low ribosomal S6 phosphorylation in WT CTL and DEN livers suggests moderate ribosomal activity. This difference was observed between control livers as well; therefore, hSDC1^+/+^ overexpression per se may result in ribosomal activation independent of hepatocarcinogenesis.

Modulation of the mTOR pathway in hSDC1^+/+^ livers had further ramifications. Akt is known to promote S22 phosphorylation and degradation of IkB which results in the release and nuclear entry of NF-κB. Akt activity was suppressed in hSDC1^+/+^ DEN livers compared to WT DEN; therefore, NF-κB p100/52 pSer869 and Rel pSer503 modifications were also reduced [44].

Further studies are needed to clarify whether and how decreased Fak Y861 activating phosphorylation hinders the development of hSDC1^+/+^ tumors, and little information is available about ERK8 pY175/177 either, albeit both have been shown to be involved in hepatocarcinogenesis [45].

One of the major alterations in WT DEN tumors was enhanced lipid metabolism, probably related to the lack of Foxo1 function [46]. The transcription factor FKHR/Foxo1 becomes inactivated and excluded from the cell nucleus when phosphorylated on S256 [47], and this was observed in WT DEN livers at month 6. Phosphorylation of Foxo1 by CDK1 at S249 has the same effect [48]. Foxo1 is a key regulator of glucose and lipid metabolism in the liver; therefore, its inactivation is a hallmark of cancer-related metabolic dysregulation [49]. Conversely, maintained action of Foxo1 was reported to protect against the disruption of lipid metabolism characteristic of cancer [50], and this was the case in our hSDC1^+/+^ DEN tumors where, in contrast to the WT DEN tumors, expression of Foxo1 remained unaltered compared to untreated controls. In hSDC1^+/+^ DEN livers, CDK1 was downregulated and the function of Foxo1 as a transcription factor was preserved. Among a plethora of its downstream effects, Foxo1 negatively regulates G1/S cell cycle transition by supporting the transcription of p21 and p27 while inhibiting the cyclins D1 and D2. Accordingly, upregulation of both cyclin-dependent kinase inhibitors was detected in hSDC1^+/+^ DEN tumors [51]. Another participant in the complex interplay is 14-3-3 zeta, a regulator of intermediary metabolism [52] recently recognized to promote cancer development by hindering Foxo1 activity [53,54]. In hSDC1^+/+^ DEN tumors, 14-3-3-zeta carried enhanced S58 phosphorylation which inhibits the formation of the active homodimer [55]. In conclusion, the net effect of changes in the mTOR pathway and related regulatory components pointed toward the inhibition of pro-oncogenic metabolism in hSDC1^+/+^ DEN livers.

### 4.6. Changes in Cell Cycle Regulation

The master transcription factor TP53 is well known to protect cells against malignant transformation [56]. TP53 activation via enhanced S392 phosphorylation was detected in hSDC1^+/+^ DEN livers at months 3 and 6. S392 phosphorylation increases the DNA binding of TP53 following DNA damage, which is the primary effect of DEN treatment [57]. This tumor suppressor effect, however, disappeared by month 9, when hepatocarcinogenesis gained momentum in hSDC1^+/+^ DEN mice. Besides S392, the phospho-tyrosine kinase array revealed additional phosphorylation of TP53 on the S15 and S46 residues (data not shown), both considered as activating phosphorylation sites of TP53 [58] that alter its DNA binding properties. Thus, in addition to Foxo1, p53 also contributes to the inhibition of cancer development in hSDC1^+/+^ DEN. Based on their overlapping targets, it has even been suggested that Foxo1 and p53 may cooperate [59]. An active role of p53 in the regulation of intermediary metabolism has been proposed [60], which involves the metabolism of carbohydrates and lipids, as well as mitochondrial functions. We have detected changes in all these aspects of metabolism in DEN-exposed livers.

### 4.7. Proteomic Analysis

Mass spectrometry revealed fundamental differences between WT and hSDC1^+/+^ samples. Dramatic upregulation of intermediary lipid metabolism was already demonstrated in the preneoplastic foci of WT DEN livers at month 3, which was completely rescued in the hSDC1^+/+^ DEN livers. Proteins implicated in lipid metabolism formed differentially regulated clusters at all time points. For now, it remains to be clarified how this phenomenon relates to the known function of SDC1 in remnant lipoprotein homeostasis [11], while the role of Fasn in hepatocarcinogenesis is well established [34,61,62].

One of the less-expected results was the occurrence of a cluster containing motor proteins in WT DEN livers as early as at month 3. The majority of these motor proteins are best known for their roles in cell motility and cytoskeletal organization; however, it appears as though they are also indispensable elements of tumorigenesis that participate in intracellular trafficking, chromosomal and centrosomal amplification, spindle formation, cell migration, and adhesion, among other functions [63]. The cluster contained myosin light and heavy chains, tropomyosin, and actin, together with a handful of regulatory proteins. The implication of motor proteins in hepatocellular carcinoma is not entirely unheard of; for example, the downregulation of myosin VI has been described to inhibit the proliferation of HCC cells [64]. In the broader context of carcinomas, elevated expression of myosin X [65] and myosin 1e were shown to promote the aggressiveness of breast cancer [66].

Interestingly, the motor protein cluster disappeared by month 6 with the exceptions of Acta and Myh4, indicating that these proteins are primarily required at the early stage of cancer development. Delayed cancer development in hSDC1^+/+^ DEN livers is neatly exemplified by the lack of this cluster at months 3 and 6 and its emergence at month 9 with essentially the same proteins as those seen in WT DEN six months earlier.

Another cluster marking carcinogenesis in the WT DEN liver was that of lipoproteins. This remained a signature alteration throughout the whole experimental period, with the number of constituents peaking at month 9. This cluster did not develop in hSDC1^+/+^ samples by the end of month 9. Likewise, hSDC1^+/+^ DEN livers enjoyed relative protection against endoplasmic reticulum stress, and did not upregulate clusterin, a well-known inducer of liver tumor metastasis [67]. Although Fasn and Acly expression decreased in WT DEN tumors over time, their levels remained higher throughout compared to hSDC1^+/+^ DEN.

The ribosome cluster of control healthy liver contained three large (10,12,31) and three small (5,24,27a) ribosomal proteins. None of them were present in hSDC1^+/+^ control samples, and only a few were present in both WT and hSDC1^+/+^ tumors. However, the number of representing proteins was consistently higher in hSDC1^+/+^ DEN livers. Interestingly, while the cluster itself persisted, its individual constituents kept changing over time, indicating the requirement for different proteins in different phases of cancer progression. Ribosomal proteins as active participants of malignant phenotype keep coming into the focus of cancer research, looking for their role in the development and progression of cancer [68,69,70]. Hyperactivation of ribosome biogenesis is suggested as a critical event in the initiation of cancer. Among others, activation of the mTORC1 complex and c-myc proto-oncogene participate in the mechanism [71,72]. However, in our experiments, the number of ribosomal proteins did not increase in the wild-type DEN exposed livers, indicating that the process is rather characterized by dysregulated ribosomal biogenesis. This notion is supported by the fact that no *Rps* was detected in WT liver tumors at month 9. A further question to address in the future is to find explanation for the enhanced ribosomal biogenesis in the control hSDC1^+/+^ livers.

Compared to WT DEN tumors, hSDC1^+/+^ DEN tumors maintained higher levels of several mitochondrial membrane electron chain proteins and preserved DHTKD1 involved in the tricarboxylic acid cycle [73]. These findings suggest a greater contribution of oxidative phosphorylation to the energy supply of hSDC1^+/+^ DEN tumors.

The collation of results from phosphokinase arrays and mass spectrometry revealed an important difference between proteins implicated in regulatory events versus those involved in intermediary metabolism and the synthesis of macromolecules. Out of more than 1000 proteins assessed by MS at each time point, hardly any signal transduction pathway components or receptor tyrosine kinases were differentially regulated, while the representation of proteins involved in intermediary metabolism or macromolecule biosynthesis increased markedly during carcinogenesis. The functions of the former are more likely to be influenced by epigenetic modifications such as phosphorylation rather than changes at the gene expression level. Intriguingly, proteoglycans including heparan sulfate proteoglycans (HSPG) seem to utilize both forms of activation. Their amounts can increase or decrease, while they can also be regulated by glycosylation or sulfation [74]. Our previous results confirmed that the differentiation of hepatoma cell lines after syndecan-1 transfection acted together with the changes of an HS pattern to a more sulfated one, as indicated by HS-specific antibodies, capable of interfering with the promoter activation of AP1 and Ets1 [16,75]. Such complexity of regulation may account, at least in part, for their often-contradictory behavior.

## 5. Conclusions

**Figure 17:**
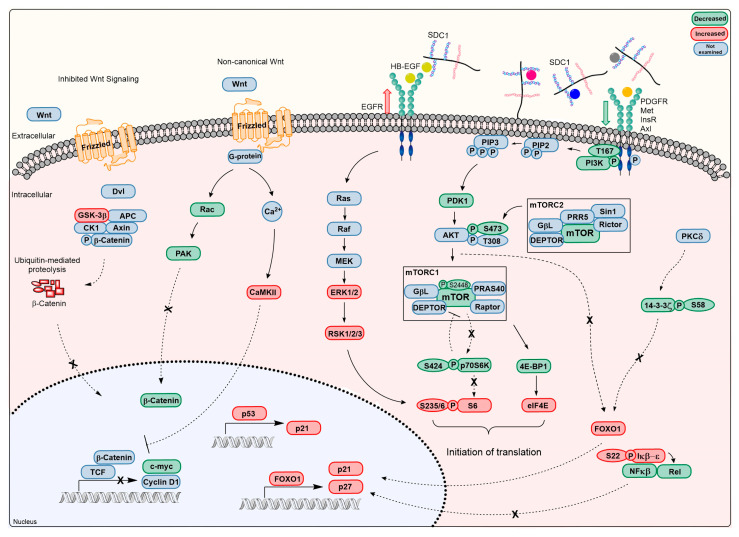
Overview of signaling pathways affected by hSDC1 overexpression in DEN-induced hepatocarcinogenesis. EGF interacts with shed SDC1 through binding to HS to create a ternary complex with EGFR that triggers its activation. Decreased activity of receptor tyrosine kinases such as MET, PDGFR, insulin receptor, and AXL, and most likely of Wnt, may result from increased SDC1 shedding, as growth factors bound to the HS chains of SDC1 are removed from the vicinity of their receptors. Consequently, the activities of PIKC3, PDK1, and Akt are downregulated. Although the mechanism of Akt pSer473 downregulation by mTORC2 requires further investigation, downregulation of insulin receptors or PDGFR are likely candidates. Impaired activity of Akt is indicated by decreased inactivating phosphorylation of GSK3, Foxo1 and IkB. The mTOR pathway is further inhibited via feedback phosphorylation of mTOR by pS6K. Upregulation of CaMKII and decrease in Rac interferes with the noncanonical pathway of β-catenin. Besides their implication in cell metabolism, both Foxo1 and p53 upregulate the CDK inhibitors p21 and p27. Together with the downregulation of c-myc and cyclinD1, the overall outcome of these events is the inhibition of cell proliferation.

SDC1, the major heparan sulfate proteoglycan of the liver, creates connections between the stroma and the intracellular compartments of the cells, and is attracting increasing attention for its ability to promote or attenuate pathological processes in a context-dependent fashion. This duality is particularly apparent in the development and progression of various cancers, because overexpression of SDC1 has been viewed as an adverse event in some tumors but beneficial in others. In this work, we aimed to dissect the roles of SDC1 in the development of liver cancer. The generation of liver-targeted hSDC1 transgenic mice provided an opportunity to analyze differences in DEN-induced hepatocarcinogenesis between WT and hSDC1^+/+^ livers (Figure 17). Following up the events of carcinogenesis over a time scale of 11 months, our observations provided robust evidence that SDC1 in this particular context exerts an oncoprotective role, albeit the modes of action were more challenging to clarify. One of our major findings was the interference of hSDC1 with the cancer-associated activation of lipid metabolism, which prevented the upregulation of more than 10 proteins involved by month 3 and facilitated the clearance of lipoproteins. In a complex interplay affecting multiple targets, overexpression of hSDC1 downregulated the mTOR and β-catenin pathways and maintained Foxo1 expression, which, together with p53, promoted the activation of p21 and p27, and hindered the action of 14-3-3 zeta. WT DEN cancers were characterized by the accumulation of lipoproteins and the loss of mitochondrial and ribosomal functions, whereas no lipoprotein accumulation was detected, and ribosomal and mitochondrial functions were maintained almost until the end of the experimental period in hSDC1^+/+^ livers when the appearance of motor proteins already signaled the onset of carcinogenesis.

## Figures and Tables

**Table 1:** Screening PCR primers for backcrossing of C57 Black animals.

Primer	Name of Primer	Sequence (5′-3′ Orientation)	T_m_ (°C)
Reverse	SJ2*	GTGGAGGCAGCTGTA	50.9
Forward	Albumin promoter	GGCAAACATACGCAAGGGA	55.8

**Table 2:** Studied mice.

Mice	Male	Female	Total
hSDC1^+/+^ transgenic DEN	22	18	40
hSDC1^+/+^ transgenic control	24	19	43
C57 Black DEN	14	12	26
C57 Black control	8	8	16

**Table 3:** Proteins involved in fat metabolism significantly overexpressed in the foci of WT DEN livers compared to hSDC1^+/+^ DEN at month 3.

Gene Name	Protein Name	Fold Change	*p*-Value
*Ehhadh*	Peroxisomal bifunctional enzyme	17.5	0.0070
*Fasn*	Fatty acid synthase	12.0	0.0012
*Acly*	ATP-citrate synthase	11.6	0.0034
*Aldh3a2*	Fatty aldehyde dehydrogenase	7.6	0.0057
*Acaca*	Acetyl-CoA carboxylase 1	5.0	0.0437
*Aadac*	Arylacetamide deacetylase	4.3	0.0002
*Aacs*	Acetoacetyl-CoA synthetase	3.9	0.0326
*Mttp*	Microsomal triglyceride transfer protein	3.9	0.0266
*Gcdh*	Glutaryl-CoA dehydrogenase	3.5	0.0185
*Apoa1*	Apolipoprotein A-I	3.5	0.0012
*Decr1*	2,4-dienoyl-CoA reductase	3.1	0.0085

**Table 4:** Differentially regulated proteins in WT DEN and hSDC1^+/+^ DEN at month 3.

Gene Family	Family Members in WT DEN Group	Family Members in hSDC1^+/+^ DEN Group
Ribosome		
*Eif*	3c; 5	3k; 3l; 4h
*Tpt*	-	1
*Rpl*	19; 38	28; 34; 35; 35a; p1
*Rps*	15; 20; 23	10; 26; 28
*Srp*	68; r	-
Proteasome		
*Psm*	d11; d14; e1	a6; b2; b6; b7; c3; d3; d4
*Adrm*	1	-
*Numb*	-	✓
Mitochondrial respiratory chain		
*Atp*	-	5j
*Cox*	-	6c
*Nduf*	-	b8; a6; a7; b7;
*Mtch*	-	2
*mt-Nd*	-	5
Myosin		
*Myoz*	1	-
*Myh*	1; 4; 7; 8	-
*Tpm*	2	-
*Myl*	1; pf	-
*Myb*	pc2	-
Actin		
*Actn*	2; 3	-
Troponin		
*Tnn*	c2; i2; t3	-
HDL formed cluster		
*Apo*	a1; a4; b	-
*Pzp*	✓	-
*Serpin*	a1b; a1d	-
*Hrg*	✓	-
*Ahsg*	✓	-
Miscellaneous	-	Elavl1; Hnrnpa0-1; Snrnp200; U2af2; Matr3; Anp32a

**Table 5:** Differentially regulated proteins in WT DEN and hSDC1^+/+^ DEN at month 9.

Gene Family	Family Members in WT DEN Group	Family Members in hSDC1^+/+^ DEN Group
Ribosome		
*Eif*	2a; 3g; 3i; 4g1; 4h	3b; 3f; 4b
*Rpl*	18a; 19; p1	13a; 27; 26; 28; 31; 34; 35a; 37a
*Rps*	-	5; 24; 26
*Eef*	-	1a2
Proteasome		
*Psm*	c3; d4; d11; d14	b5; b6; d12
*Numb*	-	✓
Mitochondrial respiratory chain		
*Atp*	-	5d; 5c1; 6v1a
*Cox*	-	7c
*Nduf*	-	b10; s4
*Mtch*	-	2
*mt-Nd*	-	5
Myosin-actin		
*Myh*	-	1; 3; 4; 7; 8; 11; 14;
*Myl*	-	1; 3; pf
*Actn*	-	2; 3
*Tnn*	-	c3
*Tpm*	-	2
*Des*	-	✓
*Atp*	-	2a1
Fat net		
*Apo*	a1; a4; c2; e; h	-
*Pzp*	✓	-
*Serpin*	a1b; a1c; c1; f2	-
*Calu*	✓	-
*Ahsg*	✓	-
*Anx*	a1; a2	-
*Ola*	1	-
*Plg*	✓	-
*Mttp*	✓	-
*Clu*	✓	-
*Kng*	1	-
mRNA splicing		
*Srsf*	1; 7	-
*Prp*	18	-
Endoplasmic retic stress—protein folding		
*Dnaj*	a2; b11; c3	-

## Data Availability

The proteomics measurement data have been submitted to the MassIVE repository under the submission number MSV000086679.

## Supplementary Materials

The following are available online at https://www.mdpi.com/article/10.3390/cancers13071548/s1, Figure S1: Desmin diagram, Figure S2: mSDC1 immunohistochemistry in hSDC1^+/+^ liver, Figure S3: Relative quantification of Fasn protein expression, Figure S4: Changes in the expression of TGF-β1, Figure S5: STRING analysis of (a) WT CTL and (b) hSDC1^+/+^ CTL liver proteomes, Figure S6: STRING analysis of (a) WT DEN and (b) hSDC1^+/+^ DEN liver proteomes at month 6, Figure S7: Raw data of Figure 7, Figure S8: Raw data of Figure 10, Figure S9: Raw data of Figure 11 (Western blot), Figure S10: Raw data of Figure 11 (WES), Figure S11: Raw data of Figure 12, Figure S12: Raw data of Figure 14, Table S1: Antibodies used, Table S2: Results of MaxQuant quantitation of lipid metabolism at month 3, Table S3: Summary of protein quantitation throughout the experimental period, Table S4: Differentially regulated proteins in WT CTL and hSDC1^+/+^ CTL, Table S5: Differentially regulated proteins in WT DEN and hSDC1^+/+^ DEN at month 6.
