# Supplementary material for: Overexpression of Human Syndecan-1 Protects against the Diethylnitrosamine-Induced Hepatocarcinogenesis in Mice
Source: Cancers (Basel). 2021 Mar 27;13(7):1548. doi: 10.3390/cancers13071548 (PMC8037268; doi:10.3390/cancers13071548)
Supplement: Supplementary file 1 [file cancers-13-01548-s001.zip › cancers-1142681-suppl-XML/Supplementary Material.docx]

Supplementary Materials：Overexpression of human syndecan-1 protects against the diethylnitrosamine-induced hepatocarcinogenesis in mice

Andrea Reszegi, Katalin Karászi, Gábor Tóth, Kristóf Rada, Lóránd Váncza, Lilla Turiák, Zsuzsa Schaff,
András Kiss, László Szilák, Gábor Szabó, Gábor Petővári, Anna Sebestyén, Katalin Dezső, Eszter Regős,
Péter Tátrai, Kornélia Baghy and Ilona Kovalszky


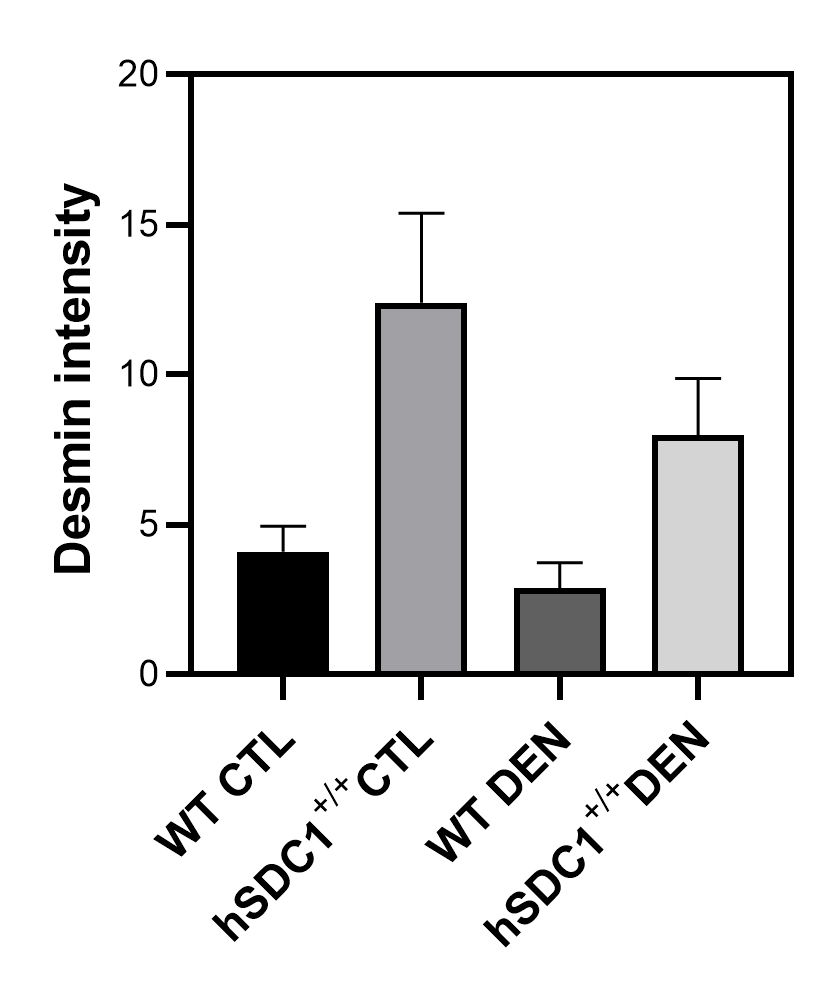


**Figure S1.** Increased expression of desmin in hSDC1^+/+^ livers compared to their WT pairs.


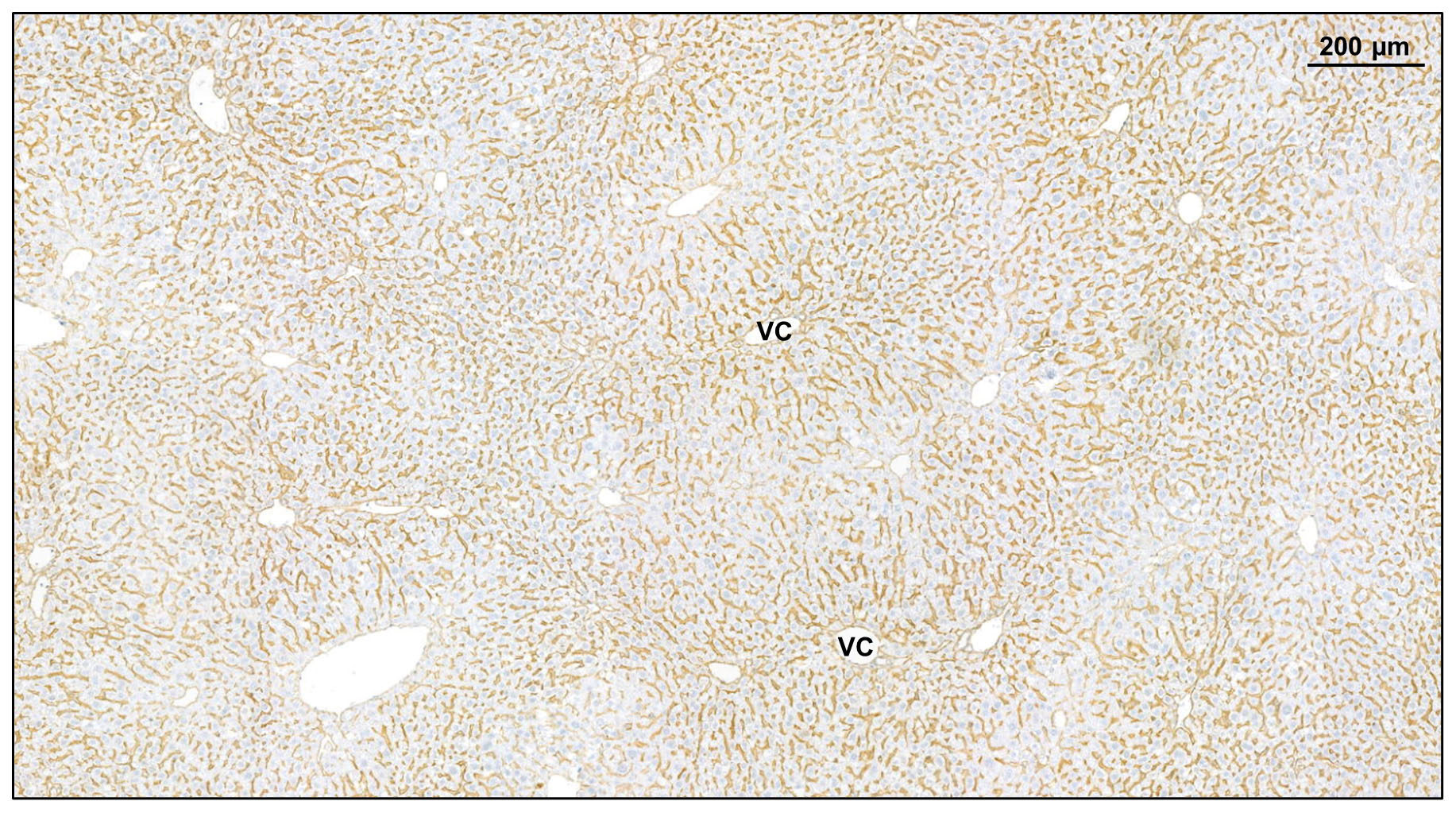


**Figure S2.** Mouse SDC1 detected in hSDC1^+/+^ mouse 3 months after DEN-exposure.


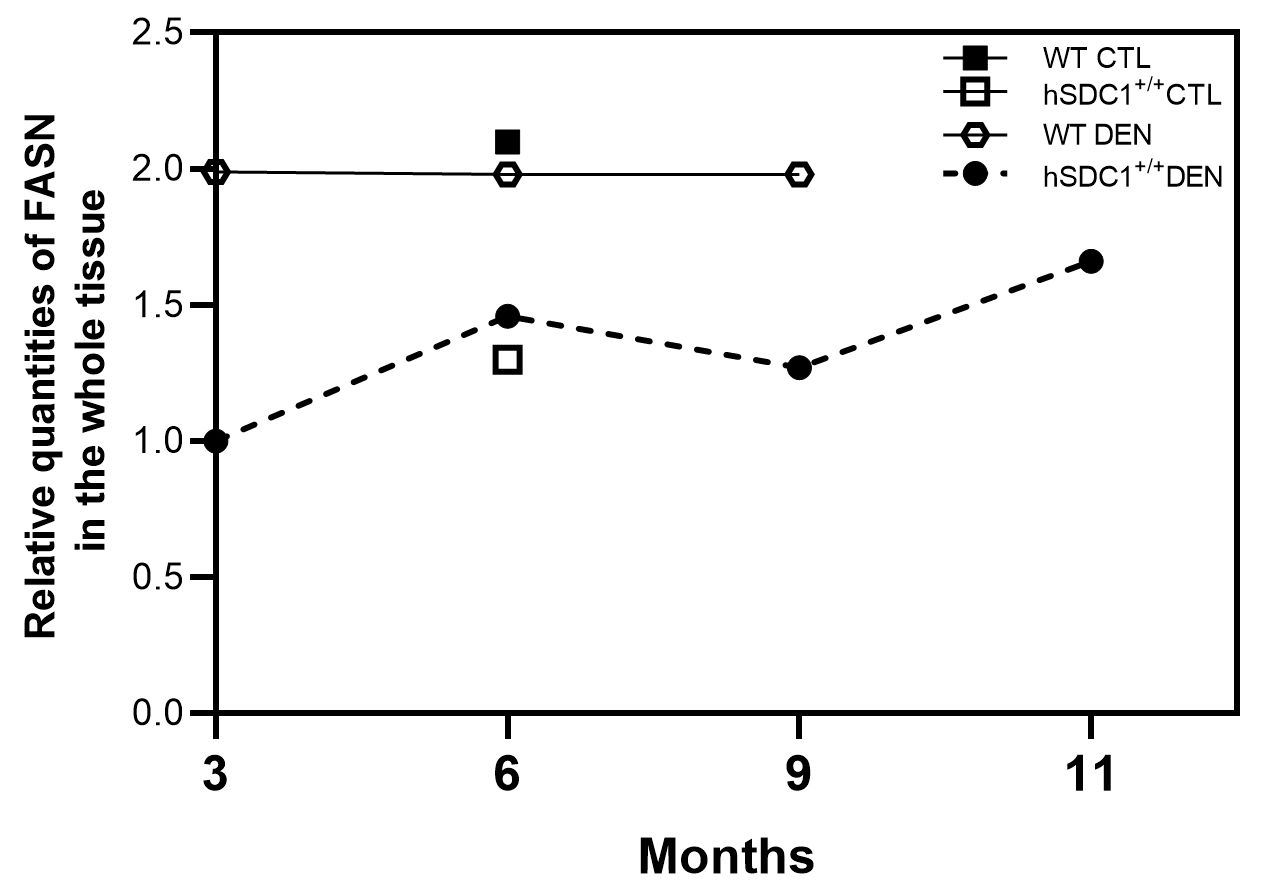


**Figure S3.** Relative quantification of Fasn protein expression by mass spectrometry in whole tissue homogenates. Fasn protein levels in homogenized whole liver tissue (including normal parenchyma along with foci / tumors, if present) were compared between WT and hSDC1^+/+^, both DEN-exposed and control, throughout the experimental period. At month 3, a 2-fold upregulation of Fasn in WT DEN relative to hSDC1^+/+^ DEN was confirmed. Fasn levels remained stably elevated in WT DEN until month 9, while in hSDC1^+/+^ DEN Fasn expression tended to increase until month 11 without ever reaching the levels of WT DEN.


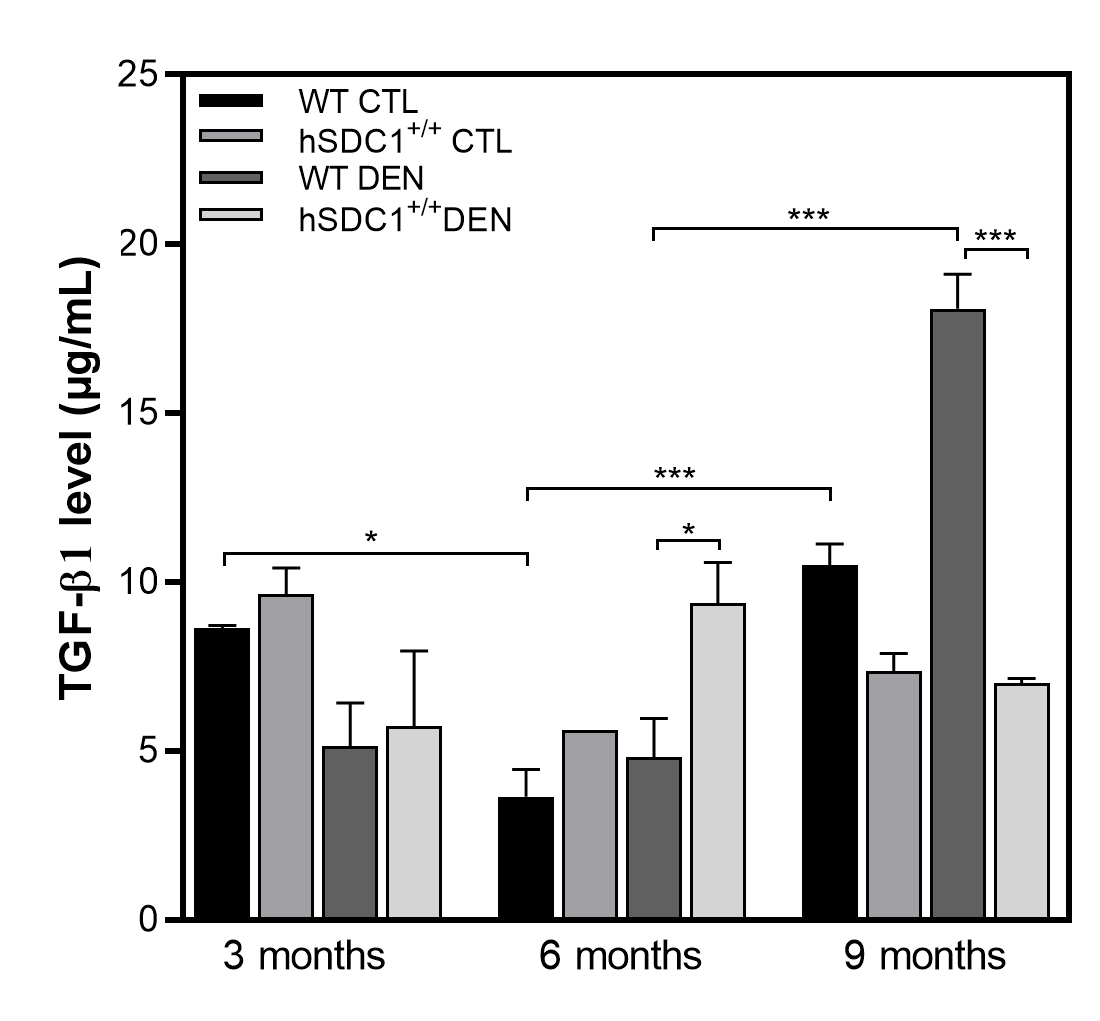


**Figure S4.** Changes in the expression of TGF-β1 across all groups from month 3 through month 9. Data points represent mean ± SD, *n* = 3; **p* < 0.05; *** *p* < 0.001.


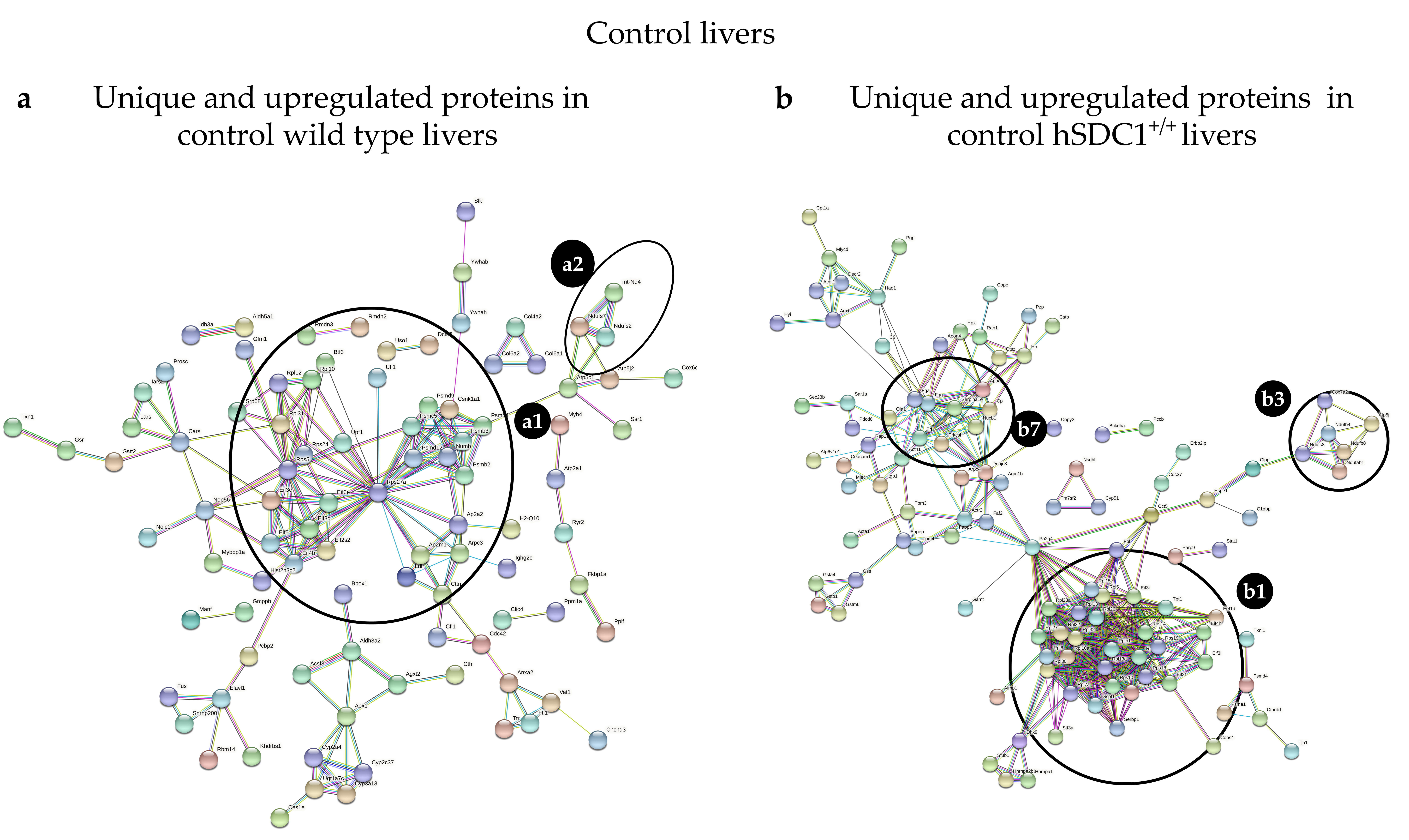


**Figure S5.** STRING analysis of (**a**) WT CTL and (**b**) hSDC1^+/+^ CTL liver proteomes. Labels: (a1) ribosomal proteins, (a2) lipoproteins and related proteins differentially regulated in WT CTL; (b1) ribosomal proteins, (b3) mitochondrial respiratory chain proteins, (b7) miscellaneous, including lipid metabolism-related proteins, proteins for G protein binding factor, and IGF binding factor, differentially regulated in hSDC1^+/+^ CTL.





**Figure S6.** STRING analysis of (**a**) WT DEN and (**b**) hSDC1^+/+^ DEN liver proteomes at month 6. Labels: (a1) ribosomal proteins, (a2) apoproteins and related, (a5) motor proteins, (a7) proteins involved in vesicular transport and membrane trafficking, differentially regulated in WT DEN; (b1) ribosomal proteins, (b3) mitochondrial respiratory chain proteins, (b4) proteasomal proteins, (b6) mRNA splicing proteins, (b7) proteins involved in vesicular transport and membrane trafficking, differentially regulated in hSDC1^+/+^ DEN.


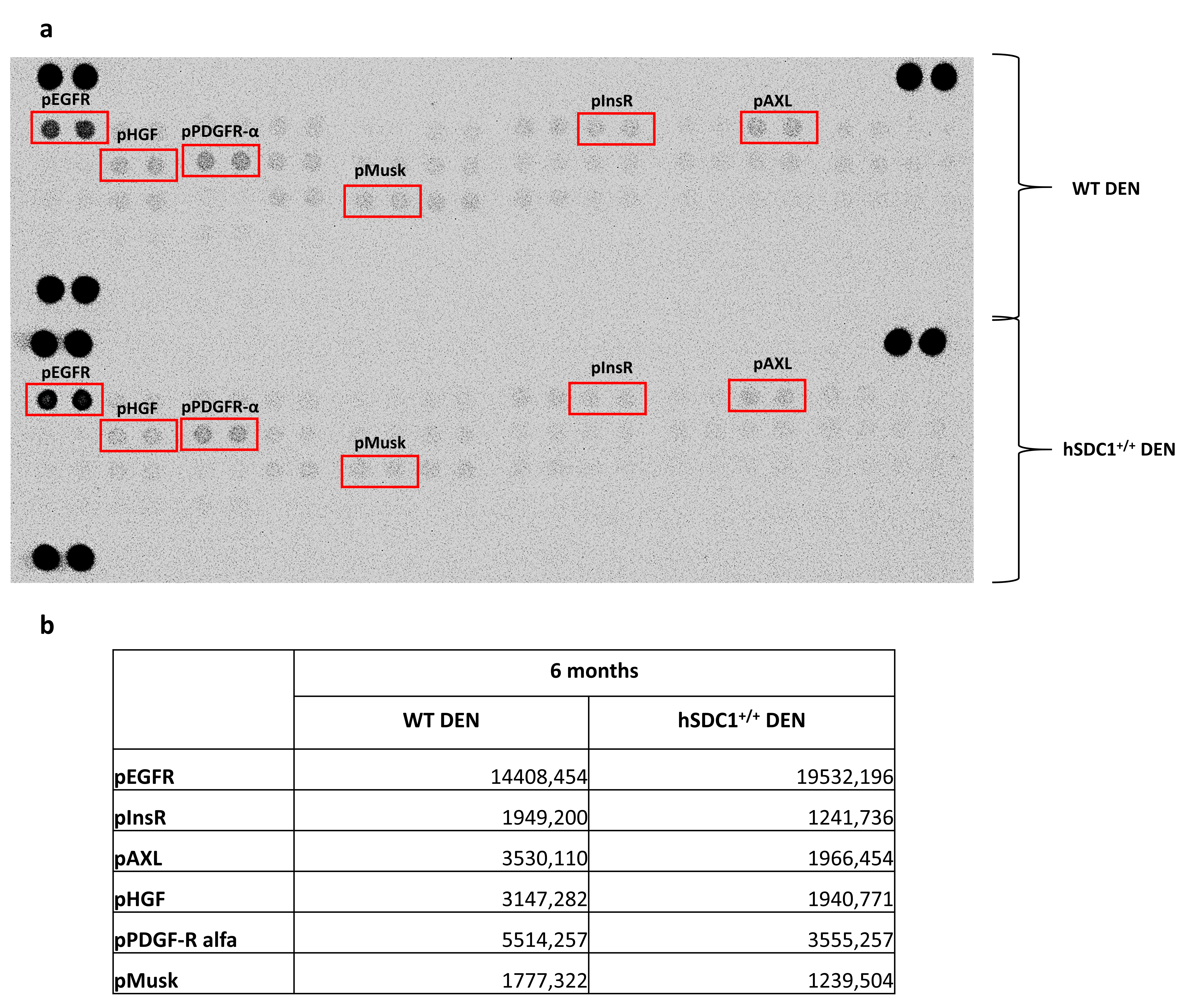


**Figure S7.** Raw data of Figure 7. (**a**) Raw image of pRTK array, (**b**) values of densitometry. Phosphorylation of EGFR, HGF, PDGFRα, Musk, InsR and AXL receptors in 6 months WT DEN vs. SDC1-transfected livers.


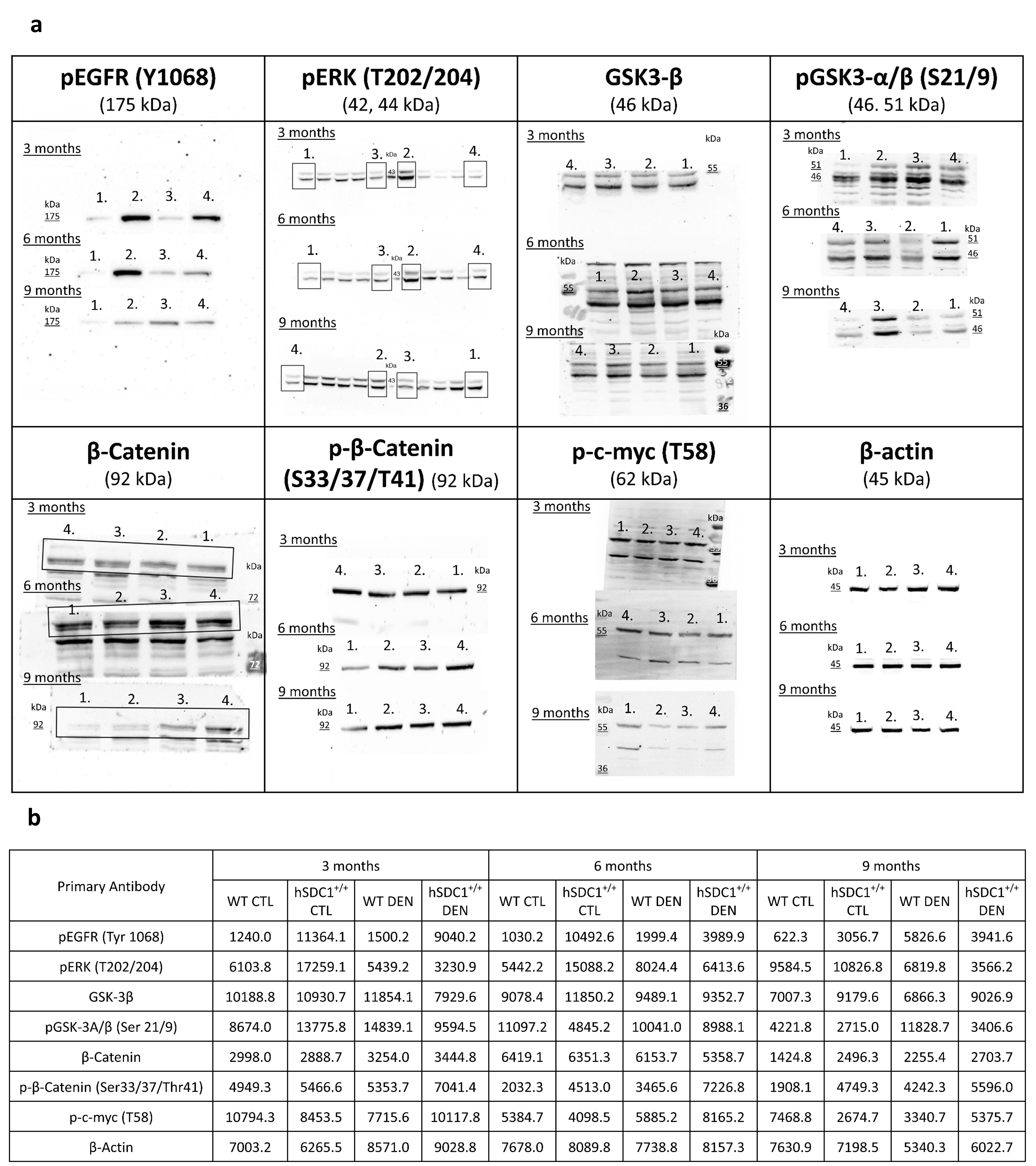


**Figure S8.** Raw data of Figure 10. (**a**) Raw Western blots, (**b**) values of densitometry. 1. WT CTL, 2. hSDC1^+/+^ CTL, 3. WT DEN, 4. hSDC1^+/+^ DEN.


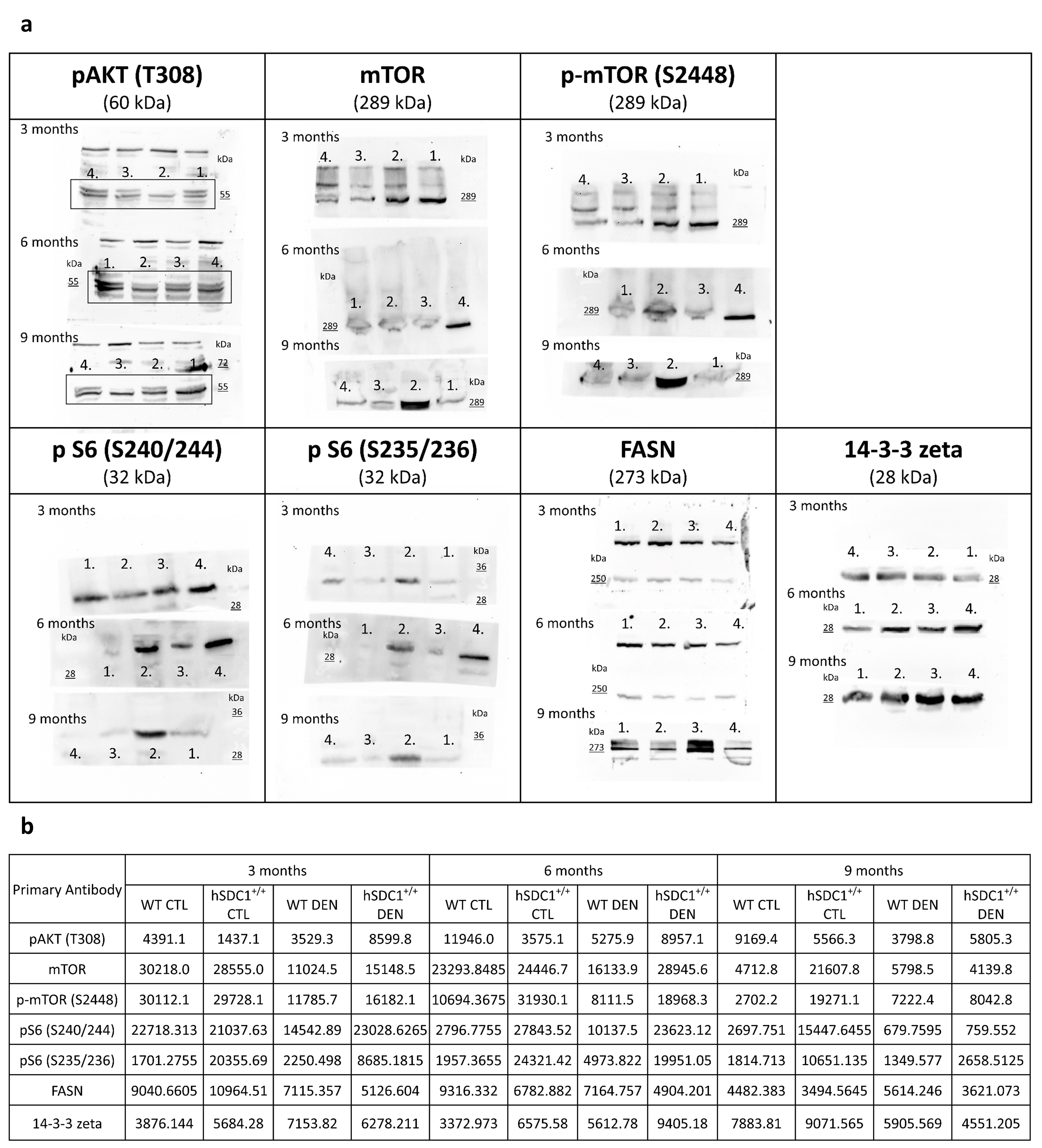


**Figure S9.** Raw data of Figure 11 (Western blot). (**a**) Raw Western blots, (**b**) values of densitometry. 1. WT CTL, 2. hSDC1^+/+^ CTL, 3. WT DEN, 4. hSDC1^+/+^ DEN.


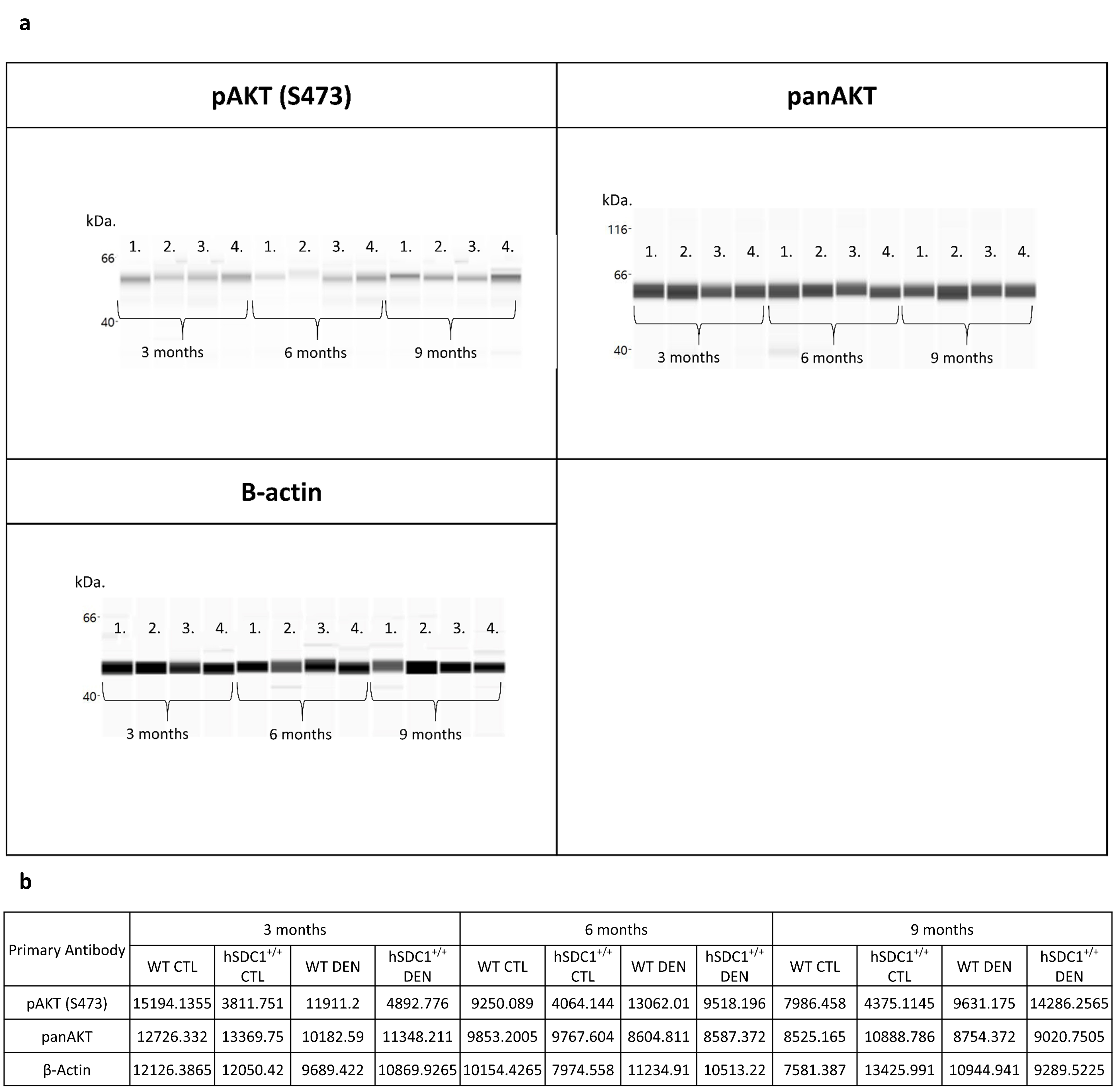


**Figure S10.** Raw data of Figure 11 (WES). (**a**) Raw Western blots, (**b**) values of densitometry. 1. WT CTL, 2. hSDC1^+/+^ CTL, 3. WT DEN, 4. hSDC1^+/+^ DEN.


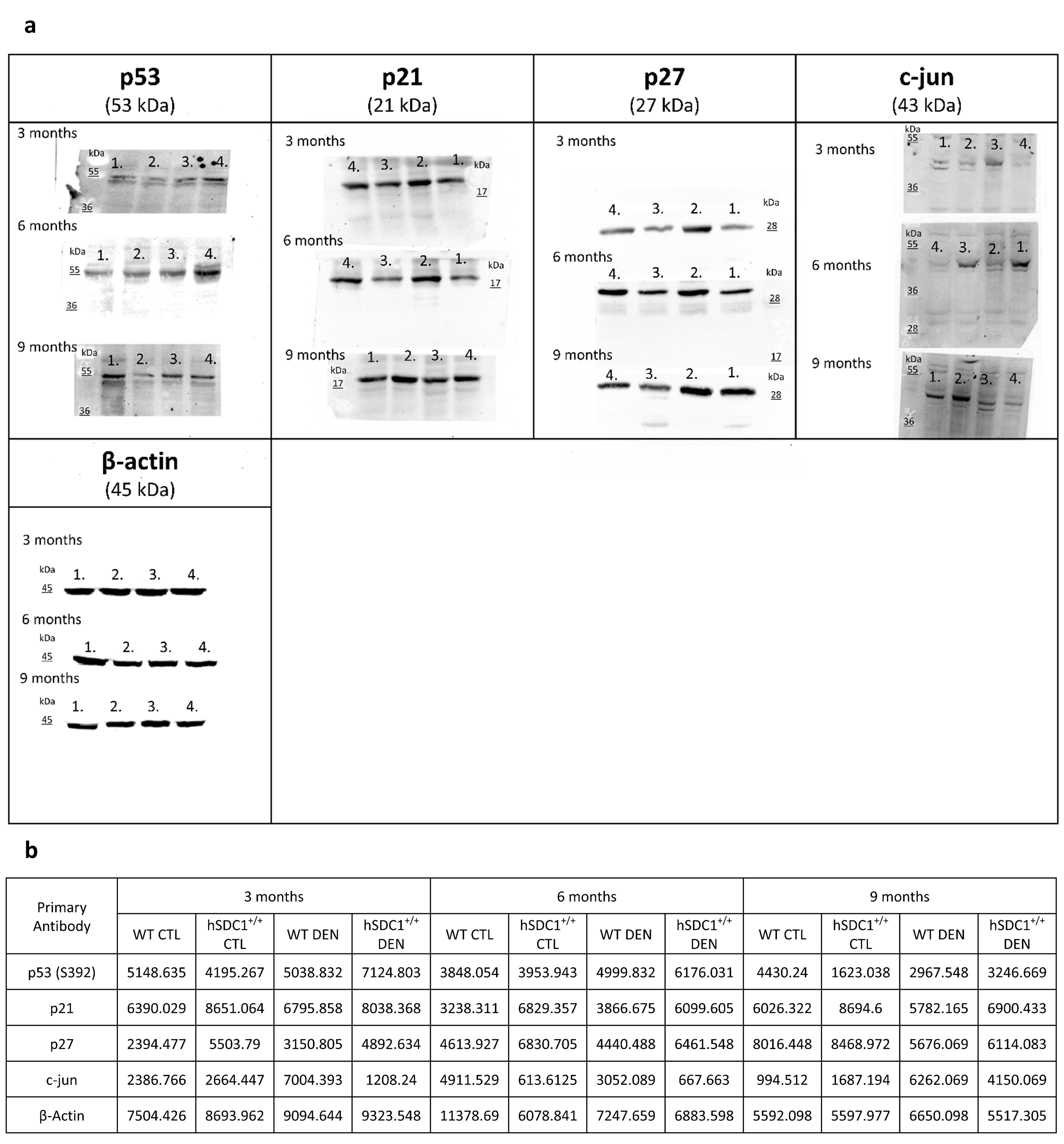


**Figure S11.** Raw data of Figure 12. (**a**) Raw Western blots, (**b**) values of densitometry. 1. WT CTL, 2. hSDC1^+/+^ CTL, 3. WT DEN, 4. hSDC1^+/+^ DEN.





**Figure S12.** Raw data of Figure 14. (**a**) Raw blot, (**b**) values of densitometry. Changes in signaling proteins detected by the Full Moon Phospho Array.

**Table S1**. Antibodies used.

| **Primary Antibody** | **Host Species, Isotype** | **Manufacturer*** | **Cat. No.** | **Dilution** | |
| --- | --- | --- | --- | --- | --- |
|  |  |  |  | **IHC#** | **blot#** |
| 14-3-3 zeta | rabbit polyclonal IgG | Invitrogen | PA5-27317 |  | 1:1000 |
| c-jun | rabbit monoclonal IgG, clone 60A8 | Cell Signaling Technology | 9165 |  | 1:1000 |
| Desmin | mouse monoclonal IgG, clone D33 | Dako | M0760 | 1:100 |  |
| FASN | rabbit monoclonal IgG, clone C20G5 | Cell Signaling Technology | 3180 | 1:300 | 1:1000 |
| GSK-3β | rabbit monoclonal IgG, clone D5C5Z | Cell Signaling Technology | 12456 |  | 1:1000 |
| human Syndecan-1 | goat polyclonal IgG | R&D Systems | AF2780 | 1:50 |  |
| MMP-14 | rabbit monoclonal IgG, clone EP1264Y | Abcam | ab51074 | 1:300 |  |
| mouse Syndecan-1 | goat polyclonal IgG | R&D Systems | AF3190 | 1:100 |  |
| mTOR | rabbit monoclonal IgG, clone 7C10 | Cell Signaling Technology | 2983 |  | 1:1000 |
| p21 | rabbit monoclonal IgG, clone EPR18021 | Abcam | ab188224 | 1:500 | 1:1000 |
| p53 | rabbit monoclonal IgG, clone EP155Y | Abcam | ab33889 |  | 1:1000 |
| pAKT (S473) | rabbit monoclonal IgG, clone 193H12 | Cell Signaling Technology | 4058 |  | 1:1000 |
| pAKT (T308) | rabbit monoclonal IgG, clone C31E5E | Cell Signaling Technology | 2965 |  | 1:1000 |
| panAKT | rabbit monoclonal IgG, clone C67E7 | Cell Signaling Technology | 4691 |  | 1:1000 |
| p-c-myc (T58) | rabbit polyclonal IgG | ThermoFisher Scientific | PA5-37654 |  | 1:1000 |
| pEGFR (Y1068) | rabbit monoclonal IgG, clone D7A5 | Cell Signaling Technology | 3777 |  | 1:1000 |
| pGSK-3α/β (S21/9) | rabbit monoclonal IgG, clone D17D2 | Cell Signaling Technology | 8566 |  | 1:1000 |
| phospho-p44/42 MAPK (Erk1/2) (T202/Y204) XP® | rabbit monoclonal IgG, clone D13.14.4E | Cell Signaling Technology | 4370 | 1:100 | 1:1000 |
| p-mTOR (S2448) | rabbit polyclonal IgG | Cell Signaling Technology | 2971 |  | 1:1000 |
| pS6 (S235/236) | rabbit monoclonal IgG, clone D57.2.2E | Cell Signaling Technology | 4858 |  | 1:1000 |
| pS6 (S240/244) | rabbit polyclonal IgG | Cell Signaling Technology | 2215 |  | 1:1000 |
| p-β-Catenin (S33/37/T41) | rabbit polyclonal IgG | Cell Signaling Technology | 9561 |  | 1:1000 |
| β-actin | mouse monoclonal IgG, clone AC-47 | Sigma-Aldrich | A2228 |  | 1:5000 |
| β-catenin | rabbit polyclonal IgG | Cell Signaling Technology | 9562 | 1:100 | 1:1000 |
| **Secondary antibody** | **Host species, isotype** | **Manufacturer*** | **Cat. No.** | **Dilution** | |
|  |  |  |  | **IHC#** | **blot#** |
| Anti-Rabbit Immunoglobulins/ HRP | goat polyclonal IgG | Dako | P0448 |  | 1:2000 |
| Anti-Mouse Immunoglobulins/ HRP | goat polyclonal IgG | Dako | P0447 |  | 1:2000 |
| Anti-Goat Immunoglobulins/ HRP | rabbit polyclonal IgG | Dako | P0449 | 1:200 | 1:2000 |

*****Cell Signaling Technology, Danvers, MA, USA; Abcam, Cambridge, UK; Dako/DakoCytomation; Invitrogen by Life Technologies, Carlsbad, CA, USA; Sigma-Aldrich Co., St. Louis, MO, USA; R&D Systems, Inc. Minneapolis, MN, USA. #**IHC**: immunohistochemistry, **blot**: Western blot

**Table S4.** Differentially regulated proteins in WT CTL and hSDC1^+/+^ CTL.

| **Gene family** | **Family members in WT Control group** | **Family members in hSDC1^+/+^ Control group** |
| --- | --- | --- |
| **Ribosome** |  |  |
| *Eif* | 2s2; 3c; 3e; 3g; 4b; 5 | 1d; 3f; 3i; 3l; 4g1 |
| *Rpl* | 10; 12; 31 | 3a; 5; 6; 7a; 10a; 13; 13a; 15; 22; 23a; 26; 27; 32; p1 |
| *Rps* | 5; 24; 27a | 10; 14; 18; 19; 25 |
| **Proteasome** |  |  |
| *Psm* | b2; b4; c5; d9; d12 | d4; e1 |
| **Mitochondrial respiratory chain** |  |  |
| *Atp* | - | 5j |
| *Cox* | - | 7a2 |
| *Nduf* | - | ab1; b4; b8; s9 |
| **Miscellaneous** | Ap2a2; Arpc3; Cytp450a4; Myh4; Ugt1a7c | Fga; Fgg; Nucb1; Prkcsh; Actn1; Ola1; Trt; Decr2; Pgp; Apoa1 |

**Table S5.** Differentially regulated proteins in WT DEN and hSDC1^+/+^ DEN at month 6

| **Gene family** | **Family members in WT DEN group** | **Family members in hSDC1^+/+^ DEN group** |
| --- | --- | --- |
| **Ribosome** |  |  |
| *Eif* | - | 3a; 3c; 3d; 3h; 3i; |
| *Rpl* | 10; 26; 34; 38 | 5; 23a; 24; 27a; 28; p1 |
| *Rps* | 26 | 6; 14; 20; 21; 24; a |
| *Mrps* | - | 7 |
| *Hnrnp* | - | ab |
| **Proteasome** |  |  |
| *Psm* | c4; d11 | a3; a5; c3; c6; d3; d4; d14 |
| **Mitochondrial respiratory chain** |  |  |
| *Atp* | - | 5f1; 5j; 5j2 |
| *Cox* | - | 5a |
| *Nduf* | - | a6; b4; b8; s; v2; |
| *Dhtk* | - | d1 |
| *mt-Nd* | - | 5 |
| **Myosin-actin** |  |  |
| *Myh* | 4 | 1; 6 |
| *Acta* | 1 | - |
| **Fat net** |  |  |
| *Apo* | a1; a1bp; e | - |
| *Pzp* | ✓ | - |
| *Serpin* | a1b; a1c; a3m; c1 | - |
| *Calu* | ✓ | - |
| *Ahsg* | ✓ | - |
| *Anx* | a2; a5 | - |
| *Rbp* | 1; 4 | - |
| *Pygb* | ✓ | - |
| *Lgal* | s1 | - |
| *Pgm* | 3 | - |
| **mRNA splicing** |  |  |
| *Srsf* |  | 3; 7 |
| *Sf* |  | 3b1 |
| *Rbm* |  | 39 |
| **Vesicular transport** |  |  |
| *Sec* | - | 13; 22b; 23b; 24a |
| *Arfga* | - | p2 |
| *Uso* | - | 1 |
| *Tmed* | - | 2; 9 |
| *Klc* | - | 4 |
| *Dync* | - | 1i2 |
| **Membrane trafficking** |  |  |
| *Rab* | - | 1; 2a; 5c; 10; 14 |
